# Explainable AI: A Review of Machine Learning Interpretability Methods

**DOI:** 10.3390/e23010018

**Published:** 2020-12-25

**Authors:** Pantelis Linardatos, Vasilis Papastefanopoulos, Sotiris Kotsiantis

**Affiliations:** Department of Mathematics, University of Patras, 26504 Patras, Greece; vasileios.papastefanopoulos@upatras.gr (V.P.); sotos@math.upatras.gr (S.K.)

**Keywords:** xai, machine learning, explainability, interpretability, fairness, sensitivity, black-box

## Abstract

Recent advances in artificial intelligence (AI) have led to its widespread industrial adoption, with machine learning systems demonstrating superhuman performance in a significant number of tasks. However, this surge in performance, has often been achieved through increased model complexity, turning such systems into “black box” approaches and causing uncertainty regarding the way they operate and, ultimately, the way that they come to decisions. This ambiguity has made it problematic for machine learning systems to be adopted in sensitive yet critical domains, where their value could be immense, such as healthcare. As a result, scientific interest in the field of Explainable Artificial Intelligence (XAI), a field that is concerned with the development of new methods that explain and interpret machine learning models, has been tremendously reignited over recent years. This study focuses on machine learning interpretability methods; more specifically, a literature review and taxonomy of these methods are presented, as well as links to their programming implementations, in the hope that this survey would serve as a reference point for both theorists and practitioners.

## 1. Introduction

Artificial intelligence (AI) had for many years mostly been a field focused heavily on theory, without many applications of real-world impact. This has radically changed over the past decade as a combination of more powerful machines, improved learning algorithms, as well as easier access to vast amounts of data enabled advances in Machine Learning (ML) and led to its widespread industrial adoption[1]. Around 2012 Deep Learning methods [2] started to dominate accuracy benchmarks, achieving superhuman results and further improving in the subsequent years. As a result, today, a lot of real-world problems in different domains, stretching from retail and banking [3,4] to medicine and healthcare [5,6,7], are tackled while using machine learning models.

However, this improved predictive accuracy has often been achieved through increased model complexity. A prime example is the deep learning paradigm, which is at the heart of most state-of-the-art machine learning systems. It allows for machines to automatically discover, learn, and extract the hierarchical data representations that are needed for detection or classification tasks. This hierarchy of increasing complexity combined with the fact that vast amounts of data are used to train and develop such complex systems, while, in most cases, boosts the systems’ predictive power, inherently reducing their ability to explain their inner workings and mechanisms. As a consequence, the rationale behind their decisions becomes quite hard to understand and, therefore, their predictions hard to interpret.

There is clear trade-off between the performance of a machine learning model and its ability to produce explainable and interpretable predictions. On the one hand, there are the so called black-box models, which include deep learning [2] and ensembles [8,9,10]. On the other hand, there are the so called white-box or glass-box models, which easily produce explainable results—with common examples, including linear [11] and decision-tree based [12] models. Although more explainable and interpretable, the latter models are not as powerful and they fail achieve state-of-the-art performance when compared to the former. Both their poor performance and the ability to be well-interpreted and easily-explained come down to the same reason: their frugal design.

**Figure 1:**
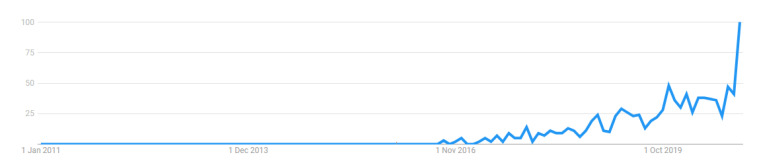
Google Trends Popularity Index (Max value is 100) of the term “Explainable AI” over the last ten years (2011–2020).

Systems whose decisions cannot be well-interpreted are difficult to be trusted, especially in sectors, such as healthcare or self-driving cars, where also moral and fairness issues have naturally arisen. This need for trustworthy, fair, robust, high performing models for real-world applications led to the revival of the field of eXplainable Artificial Intelligence (XAI) [13]—a field focused on the understanding and interpretation of the behaviour of AI systems, which. in the years prior to its revival, had lost the attention of the scientific community, as most research focused on the predictive power of algorithms rather than the understanding behind these predictions. The popularity of the search term “Explainable AI” throughout the years, as measured by Google Trends, is illustrated in Figure 1. The noticeable spike in recent years, indicating the of rejuvenation of the field, is also reflected in the increased research output of the same period.

### The Contribution of this Survey

As the demand for more explainable machine learning models with interpretable predictions rises, so does the need for methods that can help to achieve these goals. This survey will focus on providing an extensive and in-depth identification, analysis, and comparison of machine learning interpretability methods. The end goal of the survey is to serve as a reference point for both theorists and practitioners not only by providing a taxonomy of the existing methods, but also by scoping the best use cases for each of the methods and also providing links to their programming implementations–the latter being found in the Appendix A section.

## 2. Fundamental Concepts and Background

### 2.1. Explainability and Interpretability

The terms interpretability and explainability are usually used by researchers interchangeably; however, while these terms are very closely related, some works identify their differences and distinguish these two concepts. There is not a concrete mathematical definition for interpretability or explainability, nor have they been measured by some metric; however, a number of attempts have been made [14,15,16] in order to clarify not only these two terms, but also related concepts such as comprehensibility. However, all these definitions lack mathematical formality and rigorousness [17]. One of the most popular definitions of interpretability is the one of Doshi-Velez and Kim, who, in their work [15], define it as “the ability to explain or to present in understandable terms to a human”. Another popular definition came from Miller in his work [18], where he defines interpretability as “the degree to which a human can understand the cause of a decision”. Although intuitive, these definitions lack mathematical formality and rigorousness [17].

Based on the above, interpretability is mostly connected with the intuition behind the outputs of a model [17]; with the idea being that the more interpretable a machine learning system is, the easier it is to identify cause-and-effect relationships within the system’s inputs and outputs. For example, in image recognition tasks, part of the reason that led a system to decide that a specific object is part of an image (output) could be certain dominant patterns in the image (input). Explainability, on the other hand, is associated with the internal logic and mechanics that are inside a machine learning system. The more explainable a model, the deeper the understanding that humans achieve in terms of the internal procedures that take place while the model is training or making decisions. An interpretable model does not necessarily translate to one that humans are able to understand the internal logic of or its underlying processes. Therefore, regarding machine learning systems, interpretability does not axiomatically entail explainability, or vice versa. As a result, Gilpin et al. [16] supported that interpretability alone is insufficient and that the presence of explainability is also of fundamental importance. Mostly aligned with the work of Doshi-Velez and Kim [15], this study considers interpretability to be a broader term than explainability.

### 2.2. Evaluation of Machine Learning Interpretability

Doshi-Velez and Kim [15] proposed the following classification of evaluation methods for interpretability: application-grounded, human-grounded, and functionally-grounded, subsequently discussing the potential trade-offs among them. Application-grounded evaluation concerns itself with how the results of the interpretation process affect the human, domain expert, end-user in terms of a specific and well-defined task or application. Concrete examples under this type of evaluation include whether an interpretability method results in better identification of errors or less discrimination. Human-grounded evaluation is similar to application-grounded evaluation; however, there are two main differences: first, the tester in this case does not have be a domain expert, but can be any human end-user and secondly, the end goal is not to evaluate a produced interpretation with respect to its fitness for a specific application, but rather to test the quality of produced interpretation in a more general setting and measure how well the general notions are captured. An example of measuring how well an interpretation captures the abstract notion of an input would be for humans to be presented with different interpretations of the input, and them selecting the one that they believe best encapsulates the essence of it. Functionally-grounded evaluation does not require any experiments that involve humans, but instead uses formal, well-defined mathematical definitions of interpretability to evaluate quality of an interpretability method. This type of evaluation usually follows the other two types of evaluation: once a class of models has already passed some interpretability criteria via human-grounded or application-grounded experiments, then mathematical definitions can be used to further rank the quality of the interpretability models. Functionally-grounded evaluation is also appropriate when experiments that involve humans cannot be applied for some reason (e.g ethical considerations) or when the proposed method has not reached a mature enough stage to be evaluated by human users. That said, determining the right measurement criteria and metric for each case is challenging and remains an open problem.

### 2.3. Related Work

The concepts of interpretability and explainability are hard to rigorously define; however, multiple attempts have been made towards that goal, the most emblematic works being [14,15].

The work of Gilpin et al. [16] constitutes another attempt to define the key concepts around interpretability in machine learning. The authors, while focusing mostly on deep learning, also proposed a taxonomy, by which the interpretability methods for neural networks could be classified into three different categories. The first one encompasses methods that emulate the processing of data in order to create insights for the connections between inputs and outputs of the model. The second category contains approaches that try to explain the representation of data inside a network, while the last category consists of transparent networks that explain themselves. Lastly, the author recognises the promising nature of the progress achieved in the field of explaining deep neural networks, but also highlights the lack of combinatorial approaches, which would attempt to merge different techniques of explanation, claiming that such types of methods would result in better explanations.

Adadi and Berrada [17] conducted an extensive literature review, collecting and analysing 381 different scientific papers between 2004 and 2018. They arranged all of the scientific work in the field of explainable AI along four main axes and stressed the need for more formalism to be introduced in the field of XAI and for more interaction between humans and machines. After highlighting the trend of the community to explore explainability only in terms of modelling, they proposed embracing explainability in other aspects of machine learning. Finally, they suggested a potential research direction that would be towards the composition of existing explainability methods.

Another survey that attempted to categorise the existing explainability methods is this of Guidotti et al. [19]. Firstly, the authors identified four categories for each method based on the type of problem that they were created to tackle. One category for explaining black-box models, one for inspecting them, one for explaining their outcomes, and, finally, one for creating transparent black box models. Subsequently, they proposed a taxonomy that takes into account the type of underlying explanation model (explanator), the type of data used as input, the problem the method encounters, as well as the black box model that was “opened”. As with works previously discussed, the lack of formality and need for a definition of metrics for evaluating the performance of interpretability methods was highlighted once again, while the incapacity of most black-box explainability methods to interpret models that make decisions based on unknown or latent features was also raised. Lastly, the lack of interpretability techniques in the field of recommender systems is identified and an approach according to which models could be learned directly from explanations is proposed.

Upon identifying the lack of formality and ways to measure the performance of interpretability methods, Murdoch et al. [20] published a survey in 2019, in which they created an interpretability framework in the hope that it would help to bridge the aforementioned gap in the field. The Predictive, Descriptive, Relevant (PDR) framework introduced three types of metrics for rating the interpretability methods, predictive accuracy, descriptive accuracy, and relevancy. To conclude, they dealt with transparent models and post-hoc interpretation, as they believed that post-hoc interpretability could be used to elevate the predictive accuracy of a model and that transparent models could increase their use cases by increasing predictive accuracy—making clear, that, in some cases, the combination of the two methods is ideal.

A more recent study carried out by Arrieta et al. [21] introduced a different type of arrangement that initially distinguishes transparent and post-hoc methods and subsequently created sub-categories. An alternative taxonomy specifically for the deep learning interpretability methods, due to their high volume, was developed. Under this taxonomy, four categories were proposed: one for providing explanations regarding deep network processing, one in relation to the explanation of deep network representation, one concerned with the explanation of producing systems, and one encompassing hybrids of transparent and black-box methods. Finally, the authors dived into the concept of Responsible Artificial Intelligence, a methodology introducing a series of criteria for implementing AI in organizations.

## 3. Different Scopes of Machine Learning Interpretability: A Taxonomy of Methods

Different view-points exist when it comes to looking at the the emerging landscape of interpretability methods, such as the type of data these methods deal with or whether they refer to global or local properties. The classification of machine learning interpretability techniques should not be one-sided. There are exist different points of view, which distinguish and could further divide these methods. Hence, in order for a practitioner to identify the ideal method for the specific criteria of each problem encountered, all aspects of each method should be taken into consideration.

**Figure 2:**
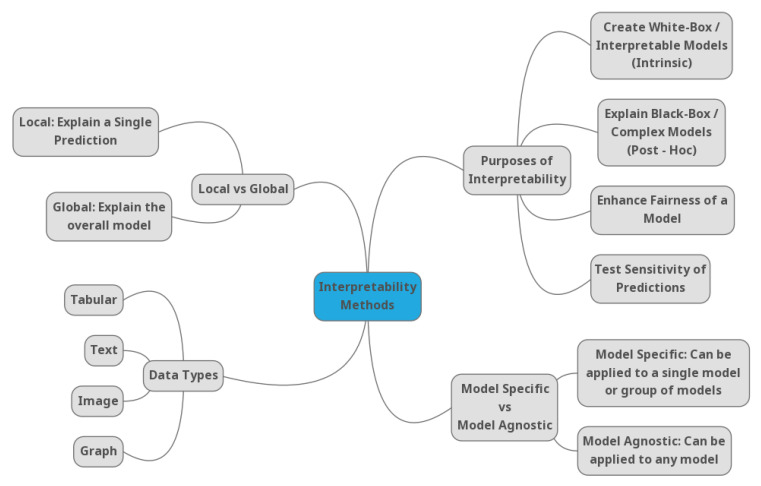
Taxonomy mind-map of Machine Learning Interpretability Techniques.

A especally important separation of interpretability methods could happen based on the type of algorithms that could be applied. If their application is only restricted to a specific family of algorithms, then these methods are called model-specific. In contrast, the methods that could be applied in every possible algorithm are called model agnostic. Additionally, one crucial aspect of dividing the interpretability methods is based on the scale of interpretation. If the method provides an explanation only for a specific instance, then it is a local one and, if the method explains the whole model, then it is global. At last, one crucial factor that should be taken into consideration is the type of data on which these methods could be applied. The most common types of data are tabular and images, but there are also some methods for text data. Figure 2 presents a summarized mind-map, which visualizes the different aspects by which an interpretability method could be classified. These aspects should always be taken into consideration by practitioners, in order for the ideal method with respect to their needs to be identified.

This taxonomy focuses on the purpose that these methods were created to serve and the ways through which they accomplish this purpose. As a result, according to the presented taxonomy, four major categories for interpretability methods are identified: methods for explaining complex black-box models, methods for creating white-box models, methods that promote fairness and restrict the existence of discrimination, and, lastly, methods for analysing the sensitivity of model predictions.

### 3.1. Interpretability Methods to Explain Black-Box Models

This first category encompasses methods that are concerned with black-box pre-trained machine learning models. More specifically, such methods do not try to create interpretable models, but, instead, try to interpret already trained, often complex models, such as deep neural networks. That is also why they sometimes are referred to as post-hoc interpretability methods in the related scientific literature.

Under this taxonomy, this category, due to the volume of scientific work around deep learning related interpretability methodologies, is split into two sub-categories, one specifically for deep learning methods and one concerning all other black-box models. For each of these sub-categories, a summary of the included methods is shown in Table 1 and Table 2 respectively.

#### 3.1.1. Interpretability Methods to Explain Deep Learning Models

The widespread adoption of deep learning methods, combined with the fact that it is in their very nature to produce black-box machine learning systems, has led to a considerable amount of experiments and scientific work around them and, therefore, tools regarding their interpretability. A substantial portion of attention regarding python tools is focused on deep learning for images more specifically on the concept of saliency in images, as initially proposed in [22]. Saliency refers to unique features, such as pixels or resolution of the image in the context of visual processing. These unique features depict the visually alluring locations in an image and a saliency map is a topographical representation of them.

Gradients: first proposed in [23], the gradients explanation technique, as its name suggests, is gradient-based attribution method, according to which each gradient quantifies how much a change in each input dimension would a change the predictions in a small neighborhood around the input. Consequently, the method computes an image-specific class saliency map corresponding to the gradient of an output neuron with respect to the input, highlighting the areas of the given image, discriminative with respect to the given class. An improvement over the initial method was proposed in [24], where the well-known Krizhevsky network [25] was utilised in order to outperform state-of-the-art saliency models by a large margin, increasing the amount of explained information by 67% when compared to state-of-the art. Furthermore, in [26], a task-specific pre-training scheme was designed in order to make the multi-context modeling suited for saliency detection.

Integrated Gradients [27] is gradient-based attribution a method that attempts to explain predictions that are made by deep neural network by attributing them to the network’s input features. It is essentially is a variation on calculating the gradient of the prediction output with respect to the features of the input, as implemented by the simpler Gradients method. Under this variation, a much desired property, which is known as completeness or Efficiency [28] or Summation to Delta [29], is satisfied: the attributions sum up to the target output minus the target output that was evaluated at the baseline. Moreover, two fundamental axioms that attribution methods ought to satisfy are identified: sensitivity and implementation invariance. Upon highlighting that most known attribution methods do not satisfy these axioms, they propose the integrated gradients method as a simple way obtain great interpretability results. Another work, closely related to the integrated gradients method, was proposed in [30], where attributions are used in order to help identify weaknesses of three question-answer models better than the conventional models, while also to provide workflow superiority.

DeepLIFT [29] is a popular algorithm that was designed to be applied on top of deep neural network predictions. The method, as described in [29], is an improvement over its first form [29], also known as the “Gradient * Input” method, where it was observed that saliency maps that were obtained using the gradient method can be greatly enhanced by multiplying the gradient with the input signal—an operation that is essentially a first-order Taylor approximation of how the output would change if the input were set to zero. The method’s superiority was demonstrated by showing considerable benefits over gradient-based methods when applied to models that were trained on natural images and genomics data. By observing the activation of each neuron, it assigns them contribution scores, calculated by comparing the difference of the output from some reference output to the differences of the inputs from their reference inputs. By optionally giving separate consideration to positive and negative contributions, DeepLIFT can also reveal dependencies that are missed by other approaches, such as the Integrated Gradients approach [27].

Guided BackPropagation [31], which is also known as guided saliency, is a variant of the deconvolution approach [32] for visualizing features learned by CNNs, which can also be applied to a broad range of network structures. Under this approach, the use of max-pooling in convolutional neural networks for small images is questioned and the replacement of max-pooling layers by a convolutional layer with increased stride is proposed, resulting in no loss of accuracy on several image recognition benchmarks.

Deconvolution, as proposed in [32], is a technique for visualizing Convolutional Neural Networks (CNNs or ConvNets) by utilising De-convolutional Networks (DeconvNets or DCNNs), as initially proposed in [33]. DeconvNets use the same components, such as filtering and pooling, but in reverse fashion: instead of mapping pixels to features, they apply the opposite. Originally, in [33] DeconvNets were proposed as a way of performing unsupervised learning; however, in [32] they are not used in any learning capacity, but rather as a tool to provide insight into the function of intermediate feature layers and pieces of information of an already trained CNN. More specifically, a novel way of mapping feature activity in intermediate layers back to the input feature space (pixels in the case of images) was proposed, showing what input pattern originally caused a given activation in the feature maps. This is done through a DeconvNet being attached to each of CNN layers, providing a continuous path back to image pixels.

Class Activation Maps, or CAMs, first introduced in [34], is another deep learning intrepretability method used for CNNs. More specifically, it’s used to indicate the discriminative regions of an image used by a CNN to identify the category of the image. A feature vector is created by computing and concatenating the averages of the activations of convolutional feature maps that are located just before the final output layer. Subsequently, a weighted sum of this vector is fed to the final softmax loss layer. Using this simple architecture, the importance of the image regions, pertaining to their classification, can, therefore, be identified by projecting back the weights of the output layer on to the convolutional feature maps. CAM has two distinct drawbacks: Firstly, in order to be applied, it requires that neural networks have a very specific structure in their final layers and, for all other networks, the structure needs to be changed and the network needs to be re-trained under the new architecture. Secondly, the method, being constrained to only visualising the final convolutional layers of a CNN, is only useful when it comes to interpreting the very last stages of the network’s image classification and it is unable to provide any insight into the previous stages.

Grad-CAM [35] is a strict generalization of CAM that can produce visual explanations for any CNN, regardless of its architecture, thus overcoming one of the limitations of CAM. As a gradient-based method, Grad-CAM uses the class-specific gradient information flowing into the final convolutional layer of a CNN in order to produce a coarse localization map of the important regions in the image when it comes to classification, making CNN-based models more transparent. The authors of Grad-CAM also demonstrated how the technique can be combined with existing pixel-space visualizations to create a high-resolution class-discriminative visualization, Guided Grad-CAM. By generating visual explanations in order to better understand image classification of popular networks while using both Grad-CAM and Guided Grad-CAM, it was shown that the proposed techniques outperform pixel-space gradient visualizations (Guided Backpropagation and Deconvolution) when evaluated in terms of localisation (the ability to localise objects in images using holistic image class labels only) and faithfulness (the ability to accurately explain the function learned by a model). While an improvement over CAM, Grad-CAM has its own limitations, the most notable including its inability to localize multiple occurrences of an object in an image, due its partial derivative assumptions, its inability to accurately determine class-regions coverage in an image, and the possible loss in signal due the continual upsampling and downsampling processes.

Grad-CAM++ [36] is an extension of the Grad-CAM method that provides better visual explanations of CNN model predictions. More specifically, object localization is extended to multiple object instances in a single image while using a weighted combination of the positive partial derivatives of the last convolutional layer feature maps with respect to a specific class score as weights to generate a visual explanation for the corresponding class label. This is especially helpful in multi-label classification problems, while the different weight assigned to each pixel makes it possible to capture the importance of each pixel separately in the gradient feature map.

Layer-wise Relevance Propagation (LRP) [37] is a “decomposition of nonlinear classifiers” technique that brings interpretability to highly complex deep neural networks by propagating their predictions backwards. The proposed propagation procedure satisfies a conservation property, whereby the magnitude of any output is remains intact, as it is backpropagated through the lower-level layers of the network: Starting from the output neurons going all the way back to the input-layer neurons, each neuron redistributes to the lower layer the same amount of information as it received from the higher layer. The method can be applied to various data types, such as images, text, and more, as well as various neural network architectures.

By pointing out and exploiting the fact that the gradient of the loss function with respect to the input can be interpreted as a sensitivity map, Smilkov et al. [38] created SmoothGrad, a method that can be applied in order to reduce noise in order visually sharpen such sensitivity maps. SmoothGrad can be combined with other sensitivity map algorithms, such as the Integrated Gradients [27] and Guided BackPropagation [31], in order to produce enhanced sensitivity maps—more specifically, two smoothing approaches were explored and experimented with: The first one, which had an excellent smoothing impact, calculates the average of maps made from many small perturbations of a given instance, while the second perturbs the data with random noise and then performs the training step. The experiments showed that these two techniques can have an additive effect, and combining them provides superior results to applying them separately. Upon performing a series of experiments, the authors conclude that the estimated smoothed gradient leads to sharper visualisations and more coherent sensitivity maps when compared to the non-smoothed gradient.

In order to interpret the predictions of deep neural networks for images, the RISE algorithm [39] creates a saliency map for any black-box model, indicating how important each pixel of the image with respect to the network’s prediction. The method follows a simple yet powerful approach: each input image is multiplied element-wise with random masks and the resulting image is subsequently fed to the model for classification. The model produces a probability-like score for the masked images with respect to each of the available classes and a saliency map for the original image is created as a linear combination of the masks. The coefficients of this linear combination are calculated while using the score that was produced by the model for the corresponding masked inputs with respect to target class.

In [42], the idea of Concept Activation Vectors (CAVs) was introduced, providing a human-friendly interpretation of a neural network internal state; an intuition of how sensitive a prediction is to a user-defined concept and how important the concept is to the classification itself. One of the issues with saliency maps is that concepts in an image, such as the “human” concept or the “animal” concept, cannot be expressed as pixels and are not in the input features either and therefore cannot be captured by saliency maps. To address this CAVs try to provide a translation between the input vector space and the high-level concept space; a CAV corresponding to a concept is essentially a vector in the direction of the values (the result of activation functions in a network’s neurons) of that concept’s set of examples. By utilising CAVs, the TCAV method provides a quantitative measure of importance of a concept if and only if the network has learned about it. Furthermore, TCAV can reveal any concept learnt, even if it was not explicitly tagged within the training set or even if was not part of the input feature set.

Yosinski et al. [40] proposed applying regularisation as an additional processing step in the saliency map creating process. More specifically, by introducing four primary regularization techniques, they enforced stronger prior distributions in order to promote bias towards more recognisable and interpretable visualisations. They showed that the best results were obtained when the different regularisers were combined, while each of these regularisation methods can also individually enhance interpretability.

In [43], an interpretability technique for neural networks operating in the natural language processing (NLP) domain was proposed. Under this approach, smaller, tailored pieces of the original input text are extracted and then used as input in order to try and produce the same output prediction as the original full-text input. These small pieces, called rationales, provide the necessary explanation and justification for the output in terms of the input. The architecture consists of two components, a generator and an encoder, which are trained to function well as a whole. The generator produces candidate rationales, and the encoder uses them to produce predicted probability scores. The generator and the encoder are trained jointly, and, through the minimization of the cost function, it is decided which candidates will be characterised as rationals. Essentially, the two components work together in order to find subsets of text that are highly associated with the predicted score.

Deep Taylor decomposition [41] is a method that decomposes a neural network’s output, for given input instance, into contributions of this instance by backpropagating the explanations from the output layer to the input. Its usefulness was demonstrated within the computer vision paradigm, in order to measure the importance of single pixels in image classification tasks; however, the method can also be applied to different types of data as both a visualization tool as well as a tool for more complex analysis. The proposed approach has strong links to relevance propagation; the theoretical connections between the Taylor decomposition of a function and rule-based relevance propagation techniques are thoroughly discussed, demonstrating a close relationship between the two approaches for a particular class of neural networks. Deep Taylor decomposition produces heatmaps, enable the user to deeply understand the impact of each single input pixel when classifying a previously unseen image. It does not require hyperparameter tuning, is robust under different architectures and datasets, and works both with custom deep network models as well as with existing pre-trained ones.

Kindermans et al. [44] showed that, while the Deconvolution [32], Guided BackPropagation [31], and LRP [37] methods help in interpreting deep neural networks, they do not produce the theoretically correct interpretation, even in the simplest neural network setting; a linear model developed while using data that were produced by a linear generative model. Using this simplified setup, the authors showed that the direction of the network’s gradient does not necessarily provide an estimate for the signal in the data, but instead corresponds to the relationship between the signal and noise; the array of parameters that are learnt by the network is in the noise-cancelling direction rather than the direction of the signal. In order to address this issue, after introducing a quality criterion for neuron-wise signal estimators in order to evaluate existing methods and ultimately obtain estimators that optimize towards this criterion, the authors propose two interpretation methods that are are theoretically sound for linear models, PatternNet and PatternAtrribution. The former is used to estimate the correct direction, improving upon the DeConvNet[32] and Guided BackPropagation[31] visualizations, while the latter to identify how much the different signal dimensions contribute to the output through the network layers. As both of the methods treat neurons independently, the produced interpretation is a superposition of the interpretations of the individual neurons.

**Figure 3:**
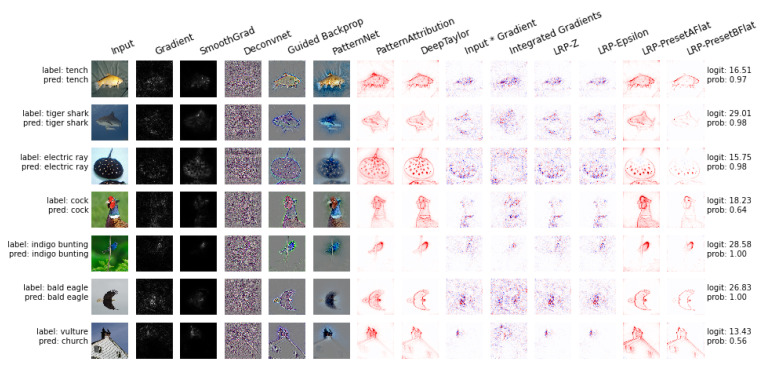
Comparison of Interpretability Methods to Explain Deep Learning Models on ImageNet sample images, using the innvestigate package.

In Figure 3, a comparison of several interpretability methods for explaining deep learning models on ImageNet sample images, while using the innvestigate package, is presented.

#### 3.1.2. Interpretability Methods to Explain any Black-Box Model

**Figure 4:**
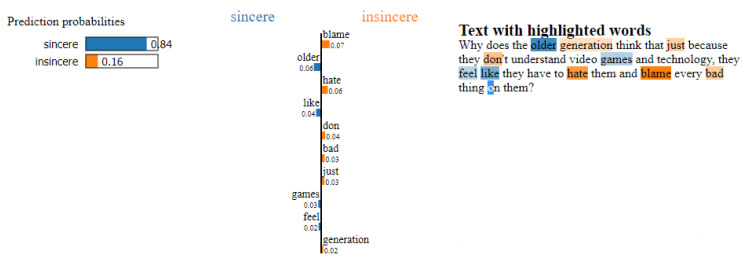
Local interpretable model-agnostic explanations (LIME) is used to explain the rationale behind the classification of an instance of the Quora Insincere Questions Dataset.

This section focuses on interpretability techniques, which can be applied to any black-box model. First introduced in [45], the local interpretable model-agnostic explanations (LIME) method is one of the most popular interpretability methods for black-box models. Following a simple yet powerful approach, LIME can generate interpretations for single prediction scores produced by any classifier. For any given instance and its corresponding prediction, simulated randomly-sampled data around the neighbourhood of input instance, for which the prediction was produced, are generated. Subsequently, while using the model in question, new predictions are made for generated instances and weighted by their proximity to the input instance. Lastly, a simple, interpretable model, such as a decision tree, is trained on this newly-created dataset of perturbed instances. By interpreting this local model, the initial black box model is consequently interpreted. Although LIME is powerful and straightforward, it has its drawbacks. In 2020, the first theoretical analysis of LIME [46] was published, validating the significance and meaningfulness of LIME, but also proving that poor choices in terms of parameters could lead LIME to missing out on important features. Figure 4 illustrates the application of the LIME method, in order to explain the rationale behind the classification of an instance of the Quora Insincere Questions Dataset.

Zafar and Khan [47] supported that the random perturbation and feature selection methods that LIME utilises result in unstable generated interpretations. This is because, for the same prediction, different interpretations can be generated, which can be problematic for deployment. In order to address this uncertainty, a deterministic version of LIME, DLIME is proposed. In this version, random perturbation is replaced with hierarchical clustering to group the data and k-nearest neighbours (KNN) to select the cluster that is believed where the instance in question belongs. Using three medical datasets among multiple explanations, they demonstrate the superiority of DLIME over LIME in terms of the Jacart Similarity.

SHAP: Shapley Additive explanations (SHAP) [48] is a game-theory inspired method that attempts to enhance interpretability by computing the importance values for each features for individual predictions. Firstly, the authors define the class of additive feature attribution methods, which unifies six current methods, including LIME [45], DeepLIFT [29], and Layer-Wise Relevance Propagation [49], which all use the same explanation model. Subsequently, they propose SHAP values as a unified measure of feature importance that maintains three desirable properties: local accuracy, missingness, and consistency. Finally, they present several different methods for SHAP value estimation and provide experiments demonstrating not only the superiority of these values in terms of differentiating among the different output classes, but also in terms of better aligning with human intuition when compared to many other existing methods.

Ancors: in [50], another model-agnostic interpretability approach that works for any black-box model with a high probability guarantee was proposed. Under this approach, high-precision, if-then rules, called anchors, are created and utilised in order to represent local, sufficient conditions for prediction. More specifically, given a prediction for an instance, an anchor is defined as a rule that sufficiently decides the prediction locally, which means that any changes to other feature values of the instance do not essentially affect the prediction value. The anchors are constructed incrementally while using a bottom-up approach. More specifically, each anchor is initialized with an empty rule, one that applies to every instance. Subsequently, in iterative fashion, new candidate rules are generated and the candidate rule with the highest estimated precision replaces the previous for that specific anchor. If, at any point, the current candidate rule meets the definition of an anchor, the desired anchor has been identified and the iterative process terminates. The authors note that this approach, attempting to discover the shortest anchor, does not directly compute and optimise towards the highest coverage. However, they highlight that such short anchors are likely to have a high coverage. By conducting a user study, the authors demonstrated that anchors not only lead to higher human precision when compared to linear explanations, but they require less effort by the user in both their application and understanding/interpretation.

Originally proposed in [51], the contrastive explanations method (CEM) is capable of generating, what the authors call, contrastive explanations for any black box model. More specifically, given any input and its corresponding prediction, the method can identify not only which features should be minimally and sufficiently present for that specific prediction to be produced, but also which features what should be minimally and necessarily absent. Many interpretation methods focus on the former part and ignore the features that are minimally, but critically, absent when trying to form an interpretation. However, according to the authors, these absent parts play an important role when it comes to forming interpretations and such interpretations are natural to humans, as demonstrated in domains, such as healthcare and criminology. Luss et al. [52] extended the CEM framework to images with much richer structure. This was achieved by defining monotonic functions that correspond to, and enable, the introduction of more concepts into an image without the deletion of any existing ones.

Wachter et al. [53] proposed a lightweight model agnostic interpretability method providing counterfactual explanations, called counterfactuals. A counterfactual explanation of a prediction describes the smallest possible change that can be applied to the feature values, so that the output prediction can be changed to a desired predefined output. The goal of this approach was not to shed light on the inner workings of a black-box system or provide insight on its decision-making, but, instead, to identify and reveal which external factors would require changing in order for the desired output to be produced. The authors highlight that counterfactual explanations, being a minimal interpretability form, are not appropriate in all scenarios and pinpoint that, in cases where the goal is the understanding of a black-box system’s functionality or the rationalisation of automated decisions, using counterfactual explanations alone may even be insufficient. Despite the downsides that are described above, counterfactual explanations can serve as an easy first step that balances between the desired properties of transparency, explainability, and accountability, as well as regulatory business interests.

Van Looveren et al. [54] underlined some problems with the previous counterfactual approach [53], most notably that it does not take local, class-specific interpretability into account, as well as that the counterfactual searching process, growing proportionally to the dimensionality of the feature space, can be computationally expensive. In order to address these issues, they proposed an improved faster, model agnostic technique for finding explainable counterfactual explanations of classifier predictions. This novel method incorporates class prototypes, constructed using either an encoder or class specific k-d trees, in the cost function to enable the perturbations to converge much faster to an interpretable counterfactual, hence removing the computational bottleneck and making the method more suitable for practical applications. In order to illustrate the effectiveness of their approach and the quality of the produced counterfactuals, the authors introduced two new metrics focusing on local interpretability at the instance level. By conducting experiments on both image data (MNIST dataset) and tabular data (Wisconsin Breast Cancer dataset), they showed that prototypes help to produce counterfactuals of superior quality. Finally, they pointed out that the perturbation of an input variable implies some notion of distance or rank among the different values of the variable; a notion that is not naturally present in categorical variables. Therefore, producing meaningful perturbations and subsequent counterfactuals for categorical features is not as straightforward. To this end, the authors proposed the use of embeddings, based on pairwise distances between the different values of a categorical variable, and empirically demonstrated the effectiveness of the proposed embeddings when combined with their method on census data.

Protodash: the work that was detailed in [55] regarding prototypes was extended in [56] by associating non-negative weightings to prototypes corresponding to their contribution, consequently creating a unifying coherent framework for both prototypes and criticisms/outliers. Moreover, under the proposed framework, since any symmetric positive definite kernel can be used, resulting in objective functions with nice properties. Subsequently, the authors propose ProtoDash, a fast, mathematically sound approximation algorithm for prototype selection that operates under the proposed framework to optimally select prototypes and learn their non-negative weights.

Permutation importance (PIMP) [57] is a heuristic approach that attempts to correct the feature importance bias through the normalisation of feature importance measures. The method, following the assumption that the random importance of a feature follows some probability distribution, attempts to estimate its parameters. This is achieved by repeatedly permuting the output array of predictions and subsequently measuring the distribution of importance for each variable on the non-permuted output. The derived p-value serves as a proxy to the corrected measure of feature importance. The usefulness of the method was demonstrated while using both simulated and real-word data to improve interpretability. As a result, an improved Random Forest (RF) model, called PIMP-RF, was proposed, which was trained on the most important features, as determined by the PIMP algorithm. PIMP can be used to complement and improve any feature-importance ranking algorithm by assigning p-values to each variable according to their permuted importance, thus improving model performance as well as model interpretability.

L2X [58] is a real-time instance-wise feature selection method that can also be used for model interpretation. More specifically, given a single training example, it tries to find the subset of its input features that are more informative in terms of the corresponding prediction for that instance. The subset is decided by a feature selector, through variational approximation, which is solely optimised towards maximising the mutual information between input features and the respective label. In the same study, a new measure called post-hoc accuracy was proposed in order to evaluate the performance of the L2X method in a quantitative way. Experiments using both real and synthetic data sets illustrate the effectiveness of the method not only in terms of post-hoc accuracy, but also terms of human-judgment evaluation, especially when it comes to nonlinear additive and feature-switching data sets.

**Figure 5:**
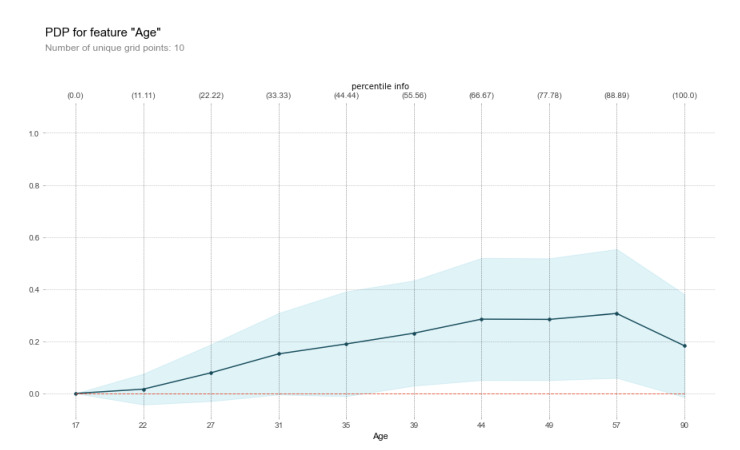
PDP of a Random Forest model, illustrating the relationship between age (feature) and income percentile (label) using the Census Income dataset (UCI Machine Learning Repository).

Friedman [59] proposed PDPs, a visualisation tool that helps to interpret any black box predictive model by plotting the impact of specific features or subsets of features on a the model’s predictions. More specifically, PDPs show how a certain set of features affects the average predicted value by marginalizing the rest of the features (its complement feature set) out. PDPs are usually very simplistic and they do not take all the different feature interactions into account. As a result, most of the time they cannot provide a very accurate approximation of the true functional relationships between the dependent and independent variables. However, they can often reveal useful information, thus greatly assisting in interpreting black box models, especially in cases where most of these interactions are of low order. Although primarily used to identify the partial relationship between a set of given features and the corresponding predicted value, PDPs can also provide visualisations for both single and multi-class problems, as well as for the interactions between features. In Figure 5, the PDP of a Random Forest model is presented, illustrating the relationship between age (feature) and income percentile (label) while using the Census Income dataset (UCI Machine Learning Repository).

Originally proposed in [60], ICE plots is a model agnostic interpretability method, which builds on the concept of PDPs and improves it. After highlighting the limitations of PDPs in capturing the complexity of the modeled relationship in the case where of substantial interaction effects are present, the authors present a refinement of he original concept. Under this innovative refinement, each plot illustrates the functional relationship between the predicted value and the feature for individual instances. As a result, given a feature, the entire distribution of individual conditional expectation functions becomes available, which enables the identification of heterogeneities and their extent.

Another method that is closely-related to PDPs is the Accumulated Local Effect (ALE) plots [61]. ALE plots trying to address the most significant shortcoming of PDPs, their assumption of independence among features, compute the conditional instead of the marginal distribution. More specifically, in order to average over other features, instead of averaging the predictions, ALE plots calculate the average differences in predictions, thus blocking the effect of correlated features.

LIVE [62] is method that is similar to LIME [45], as they both utilise surrogate models to approximate local properties of the black box models and produce coefficients of these surrogate models that are subsequently used as interpretations. However, LIVE differentiates itself from LIME in terms of local exploration as well as in terms of handling of interpretable inputs. LIVE does not create an interpretable input space by transforming the input features, but, instead, makes use of the original feature space; artificial datapoints for local exploration are generated by perturbing the datapoint in question, one feature at a time. Because the perturbed datapoints very closely match the original ones, similarities among them are measured while using the identity kernel is employed, while the original features are used as interpretable inputs.

The breakDown method, as proposed in [62], is similar to SHAP [48], as both, based on the conditioned responses of a black-box model, attempt to attribute them proportionally to the input features. However, unlike SHAP, in which the contribution of a feature is averaged over all possible conditionings, the breakDown method deals with conditionings in a greedy way, only considering a single series of nested conditionings. This approach, although not as theoretically sound as SHAP, is faster to compute and more natural in terms of interpretation.

ProfWeight: Dhurandhar et al. [63] proposed transferring knowledge from high-performing pre-trained deep neural networks to a low performing, but interpretable non-complex model to improve its performance. This was achieved by using confidence scores that are indicative of the higher level data representations that were learnt by the intermediate layers of the deep neural network, as weights during the training process of the interpretable, non-complex model.

This concludes the examination of machine interpretability methods that explain the black-box models. A diverse pool of methods, exploiting different kinds of information, have been developed, offering explanations for the different types of models as well as the different types of data, with the majority of the literature focussing on image and text data. That said, there has not been a best-in-class method developed to address every need, as most methods focus on either a specific type of model, or a specific type of data, or their scope is either local or global, but not both. Of the methods presented, SHAP is the most complete method, providing explanations for any model and any type of data, doing so at both a global and local scope. However, SHAP is not without shortcomings: The kernel version of SHAP, KernelSHAP, like most permutation based methods, does not take feature dependence into account, thus often over-weighing unlikely data points and, while TreeSHAP, the tree version of SHAP, solves this problem, its reliance on conditional expected predictions is known to produce non-intuitive feature importance values as features with no impact on predictions can be assigned an importance value that is different to zero.

### 3.2. Interpretability Methods to Create White-Box Models

This category encompasses methods that create interpretable and easily understandable from humans models. The models in this category are often called intrinsic, transparent, or white-box models. Such models include the linear, decision tree, and rule-based models and some other more complex and sophisticated models that are equally transparent and, therefore, promising for the interpretability field. This work will focus on more complex models, as the linear, decision tree and elementary rule-based models have been extensively discussed in many other scientific studies. A summary of the discussed interpretability methods to create white-box models can be found in Table 3.

Ustun and Rudin [64] proposed Supersparse Linear Integer Models (SLIM), a type of predictive system that only allows for additions, subtraction, and multiplications of input features to generate predictions, thus being highly interpretable.

In [65], Microsoft presented two case studies on real medical data, where naturally interpretable generalized additive models with pairwise interactions (GA^2^Ms), as originally proposed in [66], achieved state-of-the-art accuracy, showing that GA^2^Ms are the first step towards deploying interpretable high-accuracy models in applications like healthcare, where interpretability is of utmost importance. GA^2^Ms are generalized additive models (GAM) [67], but with a few tweaks that set them apart, in terms of predictive power, from traditional GAMs. More specifically, GA^2^Ms are trained while using modern machine learning techniques such as bagging and boosting, while their boosting procedure uses a round-robin approach through features in order to reduce the undesirable effects of co-linearity. Furthermore, any pairwise interaction terms are automatically identified and, therefore, included, which further increases their predictive power. In terms of interpretability, as additive models, GA^2^Ms are naturally interpretable, being able to calculate the contributions of each feature towards the final prediction in a modular way, thus making it easy for humans to understand the degree of impact of each feature and gain useful insight into the model’s predictions.

Boolean Rule Column Generation [68] is a technique that utilises Boolean rules, either in their disjunctive normal form (DNF) or in their conjunctive normal form (CNF), in order to create predictive models. In this case, interpretability is achieved through rule simplicity: a low number Boolean rules with few clauses and conditions in each clause can more easily be understood and interpreted by humans. The authors highlighted that most column generation algorithms, although efficient, can lead to computational issues when it comes to learning rules for large datasets, due to the exponential size of the rule-space, which corresponds to all possible conjunctions or disjunctions of the input features. As a solution, they introduced an approximate column-generation algorithm that employs randomization in order to efficiently search the rule-space and learn interpretable DNF or CNF classification rules while optimally balancing the tradeoff between classification accuracy and rule simplicity.

Generalized Linear Rule Models [69], which are often referred to as rule ensembles, are Generalized Linear Models (GLMs) [70] that are linear combinations of rule-based features. The benefit of such models is that they are naturally interpretable, while also being relatively complex and flexible, since rules are able to capture nonlinear relationships and dependencies. Under the proposed approach, a GLM is re-fit as rules are created, thus allowing for existing rules to be re-weighted, ultimately producing a weighted combination of rules.

Hind et al. [71] introduced TED, a framework for producing local explanations that satisfy the complexity mental model of a domain. The goal of TED is not to dig into the reasoning process of a model, but, instead, to mirror the reasoning process of a human expert in a specific domain, who effectively creates an domain-specific explanation system.

In summary, not a lot of progress has been made in recent years towards developing white-box models. This is most likely the result of the immense complexity modern applications require, in combination with the inherent limitations of such models in terms of predictive power—especially in computer vision and natural language processing, where the difference in performance when compared to deep learning models is unbridgeable. Furthermore, because models are increasingly expected to perform well on more than one tasks and transfer of knowledge from one domain to another is becoming a common theme, white-box models, currently being able to perform well only in a single task, are losing traction within the literature and they are dropping further behind in terms of interest.

### 3.3. Interpretability Methods to Restrict Discrimination and Enhance Fairness in Machine Learning Models

Because machine learning systems are increasingly adopted in real life applications, any inequities or discrimination that are promoted by those systems have the potential to directly affect human lives. Machine Learning Fairness is a sub-domain of machine learning interpretability that focuses solely on the social and ethical impact of machine learning algorithms by evaluating them in terms impartiality and discrimination. The study of fairness in machine learning is becoming more broad and diverse, and it is progressing rapidly. Traditionally, the fairness of a machine learning system has been evaluated by checking the models’ predictions and errors across certain demographic segments, for example, groups of a specific ethnicity or gender. In terms of dealing with a lack of fairness, a number of techniques have been developed both to remove bias from training data and from model predictions and to train models that learn to make fair predictions in the first place. In this section, the most widely-used machine learning fairness methods are presented, discussed and finally summarised in Table 4.

Disparate impact testing [72] is a model agnostic method that is able to assess the fairness of a model, but is not able to provide any insight or detail regarding the causes of any discovered bias. The method conducts a series of simple experiments that highlight any differences in terms of model predictions and errors across different demographic groups. More specifically, it can detect biases regarding ethnicity, gender, disability status, marital status, or any other demographic. While straightforward and efficient when it comes to selecting the most fair model, the method, due to the simplicity of its tests, might fail to pick up on local occurrences of discrimination, especially in complex models.

A way to ensure fairness in machine learning models is to alter the model construction process. In [73], three different data preprocessing techniques to ensure fairness in classification tasks are analysed. The first presented technique, which is called suppression, detects the features that correlate the most, according to some threshold with any sensitive features, such as gender or age. In order to diminish the impact of the sensitive features in the model’s decisions, the sensitive features along with their most correlated features are removed before training. This forces the model to learn from and, therefore, base its decisions on other attributes, thus not being biased against certain demographic segments. The second technique is called “massaging the dataset” and it was originally proposed in [74]. In order to remove the discrimination from the input data, according to this technique, some relabelling is applied to of some instances in the dataset. First, using a ranker, the instances most likely to be victims (discriminated ones) or most likely to be profiters (favoured ones) are detected, according to their probability of belonging to the corresponding class without taking the sensitives attributes into account. Subsequently, their labels are changed and a classifier is trained on this modified data that is free of bias. Finally the idea behind the third preprocessing technique, as initially presented in [75], is to apply different weights to the instances of the dataset based on frequency counts with respect to the sensitive column. The weight of an instance is calculated as the expected probability that its sensitive feature value and class appear together, while assuming that they are independent, divided by the respective observed probability. Reweighing has a similar effect to the “massaging” approach, but its major advantage is that it does alter the labels of the dataset. Similarly to disparate impact testing, the described data preproecessing methods might fail to pick up on local occurrences of discrimination, especially in complex models.

Another data preprocessing technique for removing the bias from machine learning models was proposed in [76]. More specifically, having the following three goals in mind: controlling discrimination, limiting distortion in individual instances, and preserving utility, the authors derived a convex optimization for learning a data representation that captures these goals.

Adversarial debiasing [77] is a framework for mitigating biases concerning demographic segments in machine learning systems by selecting a feature regarding the segment of interest and, subsequently, training a main model and an adversarial model simultaneously. The main model is trained in order to predict the label, whereas the adversarial model based on the main model’s prediction for each instance tries to predict the demographic segment of the instance; the objective is to maximize the main model’s ability to correctly predict the label, while, at the same time, minimizing the adversarial model’s ability to predict the demographic segment in question. Adversarial debiasing can be applied to both regression and classification tasks, regardless of the complexity of the chosen model. With regards to the sensitive features of interest, both continuous and discrete values can be handled and any imposed constraints can enforced across multiple definitions of fairness.

Kamiran et al. [78] pointed out that many of the methods that make classifiers aware of discriminatory biases require data modifications or algorithm tweaks and they are not very flexible with respect to multiple sensitive feature handling and control over the performance vs. discrimination trade-off. As a solution to these problems, two new methods methods that utilise decision theory in order to create discrimination-aware classifiers were proposed, namely Reject Option based Classification (ROC) and Discrimination-Aware Ensemble (DAE), neither of which require any data preprocessing or classifier adjustments. ROC can be viewed as a cost-based classification method, in which misclassifying an instance of a non-favoured group as negative results is much higher punishment than wrongly predicting a member of a favored group as negative. DAE employs an ensemble of classifiers. Ensembles, by nature, can be very useful in reducing bias. According to the authors, this is because the greater the number of classifiers and the more diverse these classifiers are, the higher the probability that some of them will be fair. Under this assumption, the discrimination awareness of such an ensemble can be controlled by adjusting the diversity of its voting classifiers, while the trade-off between accuracy and discrimination in DAEs depends on the disagreements between the voting classifiers and number of instances that are incorrectly classified.

Liu et al. [79] highlighted that most work in machine learning fairness had mostly studied the notion of fairness within static environments, and it had not been concerned with how decisions change the underlying population over time. They argued that seemingly fair decision rules have the potential to cause harm to disadvantaged groups and presented the case of loan decisions as an example where the introduction of seemingly fair rules can all decrease the credit score of the affected population over time. After emphasising the importance of temporal modelling and continuous measurement in evaluating what is considered fair, they concluded that in order for fairness rules to be set, rather than just considering what seems to be fair at a stationary point, an approach that takes the long term effects of such rules on the population in dynamic fashion into consideration is needed.

The problem of algorithmically allocating resources when in shortage was studied in [80] and, more specifically, the notion of fairness within this procedure in terms of groups and the potential consequences. An efficient learning algorithm is proposed that converges to an optimal fair allocation, even without any prior knowledge of the frequency of instances in each group; only the number of instances that received the resource in a given allocation is known, rather than the total number of instances. This can be translated to the fact that the creditworthiness of individuals not given loans is not known in the case of loan decisions or to the fact that some crimes committed in areas of low policing presence are not known either. As an application their framework, the authors considered the predictive policing problem, and experimentally evaluated their proposed algorithm on the Philadelphia Crime Incidents dataset. The effectiveness of the proposed method was proven, as, although trained on arrest data that were produced by its own predictions for the previous days, potentially resulting in feedback loops, the algorithm managed to overcome them.

Feedback loops in the context of predictive policing and the allocation of policing resources were also studied in [81]. More specifically, the authors first highlighted that feedback loops are a known issue in predictive policing systems, where a common scenario includes police resources being spent repeatedly on the same areas, regardless of the true crime rate. Subsequently, they developed a mathematical model of predictive policing which revealed the reasons behind the occurrence of feedback loops and showed that a relationship exists between the severity of problems that are caused by a runaway feedback loop and the variance in crime rates among area. Finally, upon acknowledging that incidents reported by citizens can alleviate the impact of runaway feedback, the authors demonstrated ways of altering the model inputs, though which predictive policing systems, which are able to overcoming the runaway feedback loop and, therefore, capable of learning the true crime rate, can be produced.

Models of strategic manipulation is a category of models that attempt to capture the dynamics between a classifier and agents in an environment, where all of the agents are capable, to the same degree, of manipulating their features in order to deceit the classifier into making a decision in their favour. In real world social environments, however, an individual’s ability to adapt to an algorithm does not merely relate to their personal benefit of getting a favourable decision, instead it heavily depends on a number of complex social interactions and factors within the environment. In [82] strategic manipulation models were studied and adapted in an environment of social inequality, in which different social groups have to pay different costs of manipulation. It was proven that, in such a setting, a decision making model exhibited a behaviour where some members of the advantaged group incorrectly received a favourable decision, while some members of the disadvantaged group incorrectly received a non-favourable one. The results also demonstrated that any tools attempting to evaluate an individual’s fitness or eligibility can potentially have harmful social consequences when the individuals’ capacities to adaptively respond differ. Finally, the authors conclude that the increasing use of decision-making machine learning tools in our imperfect social world will require the design of suitable models and the development of a sound theoretical background that would explicitly address critical issues, such as social stratification and unequal access, in order for true fairness to be achieved.

Milli et al. [83] also studied how individuals adjust their behaviour strategically to manipulate decision rules in order to gain favourable treatment from decision-making models. They reiterated that the design of more conservative decision boundaries in an effort to enhance robustness of decision making systems against such forms of distributional shift is significantly needed in order for fairness to be achieved. However, the authors showed, through experimentation, that although stricter decision boundaries add benefit to the decision maker, this is done at the expense of the individuals being classified. There is, therefore, some trade-off between the accuracy of the decision maker and the impact to the individuals in question. More specifically, a notion of “social burden” was introducedin order to quantify the cost of strategic decision making as the expected cost that a positive individual needs to meet to be correctly classified as positive, and it was proven that any increase in terms of the accuracy of the decision maker necessarily corresponds to an increase in the social burden for the individuals. Lastly, they empirically demonstrated that any extra costs occurring for individuals have the potential to be disproportionately harmful towards the already disadvantaged groups of individuals, highlighting that any strategy towards more accurate decision making must also weigh in social welfare and fairness factors.

Counterfactual fairness, which is defined strictly in [84], attempts to capture the intuition that a decision affecting an individual is fair if it would affect the same individual in the same way both the actual world and in a counterfactual world, where the individual would be a member of a different demographic group. In the same study, it was argued that it was crucial for causality in fairness to be addressed and subsequently a framework for modeling fairness using tools from causal inference was proposed. According to the authors, any measures of causality in fairness measures should not only consist of quantities free of counterfactuals, but is also essential that counterfactual causal guarantees are pursued. The proposed framework, which is based on the idea of counterfactual fairness, allows for the users to produce models that, instead of merely ignoring sensitive attributes that potentially reflect social biases towards individuals, are able to take such features into consideration and compensate for them accordingly.

The fairness of word embeddings, a vectorised representation of text data, used in many real world machine learning application, was studied in [85] and it was revealed that word embeddings, even those that were trained on Google News articles, carry strong gender bias. More specifically, two very useful, in terms of embedding debiasing, properties were shown. Firstly, it was shown that there exists a direction in the embedding space towards which gender stereotypes can be captured. Secondly, it was shown that gender neutral words can be linearly separated from gender definition words in the embedding space. Subsequently, metrics for quantifying both the direct and indirect gender stereotypes present in the word embeddings were created and an algorithm that utilises the previous two properties and tweaks the embedding vectors in order for gender bias to be removed was proposed by the authors.

According to [86], fairness should be realised not only segment-wise, but also at an individual level. In order to achieve this, fairness was formulated into a data representation problem, where any representations learnt would need to be optimised towards two competing objectives: similar individuals should have similar encodings; however, such encodings should be ignorant of any sensitive information regarding the individual.

In [87], three approaches for making the naive Bayes classifier discrimination-free were proposed. The first approach was to regulate the conditional probability distribution of the sensitive feature values given that the label is positive, by simply boosting the probability of the disadvantaged sensitive feature values given the positive label, while, at the same time, decreasing the probability of the favoured sensitive feature values given the positive label. While easy to follow and implement, this approach brings the downside of either reducing or boosting the number of positive labels that are produced by the model, depending on the difference between the frequency of the favoured sensitive values and frequency of the discriminated sensitive values in the input data. The second approach involves training a different model for every sensitive attribute value. The case where a sensitive feature has two values, and, therefore, two models were trained, was illustrated: one model was developed using only the rows that had a favoured sensitive value, while another model only utilised the rows that had a discriminated sensitive value. The different models are part of a meta-model, where discrimination is mitigated by adjusting the conditional probability, as described in the first approach. In the third approach, a latent variable is introduced to the modelling procedure, which corresponds to the non-biased label and the model parameters were optimized towards likelihood-maximisation while using the expectation-maximization (EM) algorithm.

In [88], a framework for fair classification, which consisted of two parts, was presented. More specifically, the first part involves the development of a task-specific metric in order to evaluate the degree of similarity among individuals with respect to the classification task, whereas the second part consists of an algorithmic procedure that is capable of maximizing the objective function, subject to the fairness constraint, according to which, similar decisions should be made for similar individuals. Furthermore, the framework was adjusted towards the much related goal of guaranteeing statistical parity, while, as previously, ensuring that similar individuals are provided with analogous decisions. Finally, the close relationship between privacy and fairness was discussed and, more specifically, how fairness can be further promoted using tools and approaches developed within the framework of differential privacy.

The difference between the fairness of the decision making process, also known as procedural fairness, and the fairness of the decision outcomes, also known as distributive fairness, was brought up by the authors of [89], who also emphasised that the majority of the scientific work on machine learning fairness revolved around the latter. For this gap to be bridged, procedural fairness metrics were introduced in order for the impact of input features used in the decision to be taken into consideration and for the moral judgments of humans regarding the use of these features to be quantified.

Building on from [90], where the concept of meritocratic fairness was introduced, Kearns et al. [91] performed a more comprehensive analysis on the broader issue of realising superior guarantees in terms of performance, while relaxing the model assumptions. Furthermore, the issue of fairness in infinite linear bandit problems was studied and a scheme for meritocratic fairness regarding online linear problems was produced, which was significantly more generic and robust than the existing methods. Under this scheme, fairness is satisfied by ensuring optimality in terms of reward: no actions that lead to preferential treatments are taken, unless the algorithm is certain that the reward of such an action would be higher reward. In practice, this is achieved by calculating confidence intervals around the expected rewards for the different individuals and, based on this process, two individuals are said to be linked if their corresponding confidence intervals are overlapping, and chained if they can reach each other through a chain of intermediate linked individuals.

The fact that the majority of notions or definitions of machine learning fairness merely focus on predefined social segments was criticised in [96]. More specifically, it was highlighted that such simplistic constraints, while forcing classifiers to achieve fairness at segment-level, can potentially bring discrimination upon sub-segments that consist of certain combinations of the sensitive feature values. As a first step towards addressing this, the authors proposed defining fairness across an exponential or infinite number of sub-segments, which were determined over the space of sensitive feature values. To this end, an algorithm that produces the most fair, in terms of sub-segments, distribution over classifiers was proposed. This is achieved by the algorithm through viewing the sub-segment fairness as a zero-sum game between a Learner and an Auditor, as well as through a series of heuristics.

Following up from other studies demonstrating that the exclusion of sensitive features cannot fully eradicate discrimination from model decisions, Kamishima et al. [99] presented and analysed three major causes of unfairness in machine learning: prejudice, underestimation, and negative legacy. In order to address the issue of indirect prejudice, a regulariser that was capable of restricting the dependence of any probabilistic discriminative model on sensitive input features was developed. By incorporating the proposed regulariser to logistic regression classifiers, the authors demonstrated its effectiveness in purging prejudice.

In [92], a framework for quantifying and reducing discrimination in any supervised learning model was proposed. First, an interpretable criterion for identifying discrimination against any specified sensitive feature was defined and a formula for developing classifiers that fulfil that criterion was introduced. Using a case study, the authors demonstrated that, according to the defined criterion, the proposed method produced the Bayes optimal non-discriminating classifier and justified the use of postprocessing over the altering of the training process alternative by measuring the loss that results from the enforcement of the non-discrimination criterion. Finally, the potential limitations of the proposed method were identified and pinpointed by the authors, as it was shown that not all dependency structures and not all other proposed definitions or intuitive notions of fairness can be captured while using the proposed criterion.

Pleiss et al. [97], building on from [92], studied the problem of producing calibrated probability scores, the end goal of many machine learning applications, while, at the same time, ensuring fair decisions across different demographic segments. They demonstrated, through experimentation on a diverse pool of datasets, that probability calibration is only compatible with cases where fairness is pursued with respect to a single error constraint and concluded that maintaining both fairness and calibrated probabilities, although desirable, is often nearly impossible to achieve in practice. For the former cases, a simple postprocessing technique was proposed that calibrates the output scores, while, at the same time, maintaining fairness by suppressing the information of randomly chosen input features.

Celis et al. [98] highlighted that, although efforts have been made in recent studies to achieve fairness with respect to some particular metric, some important metrics have been ignored, while some of the proposed algorithms are not supported by a solid theoretical background. To address these concerns, they developed a meta-classifier with strong theoretical guarantees that can handle multiple fairness constraints with respect to multiple non-disjoint sensitive features, thus enabling the adoption and employment of fairness metrics that were previously unavailable.

In [94], a new metric for evaluating decision boundary fairness both in terms of disparate treatment and disparate impact at the same time, with respect to one or more sensitive features was introduced. Furthermore, utilising this metric, the authors designed a framework comprising of two contrasting formulations: the first one optimises for accuracy subject to fairness constraints, while the second one optimises towards fairness subject to accuracy constraints. The proposed formulations were implemented for logistic regression and support vector machines and evaluated on real-world data, showing that they offer fine-grained control over the tradeoff between the degree of fairness and predictive accuracy.

Following up from their previous work [94], Zafar et al. [93] introduced a novel notion of unfairness, which was defined through the rates of misclassification, called disparate mistreatment. Subsequently, they proposed intuitive ways for measuring disparate mistreatment in classifiers that rely on decision boundaries to make decisions. By experimenting on both synthetic and real world data, they demonstrated how easily the proposed measures can be converted into optimisation constraints, thus incorporated in the training process, and how well they work in terms of reducing disparate mistreatment, while maintaining high accuracy standards. However, they warned of the potential limitations of their method due to the absence of any theoretical guarantees on the global optimality of the solution as well as due to the the approximation methods used, which might prove to be inaccurate when applied to small datasets.

In another work by Zafar et al. [100], it was pointed out that many of the existing notions of fairness, regarding treatment or impact, are too rigorous and restrictive and, as a result, tend to hinder the overall model performance. In order to address this, the authors proposed notions of fairness that are based on the collective preference of the different demographic groups. More specifically, their notion of fairness tries to encapsulate which treatment or outcome would the different demographic groups prefer when given a list of choices to pick from. For these preferences to be taken into consideration, proxies that capture and quantify them were formulated by the authors and boundary-based classifiers were optimised with respect to these proxies. Through empirical evaluation, while using a variety of both real-world and synthetic datasets, it was illustrated that classifiers pursuing fairness based on group preferences achieved higher predictive accuracy than those seeking fairness through strictly defined parity.

Agarwal et al. [95] introduced a systematic framework that incorporates many other previously outlined definitions of fairness, treating them as special cases. The core concept behind the method is to reduce the problem of fair classification to a sequence of fair classification sub-problems, subject to the given constraints. In order to demonstrate the effectiveness of the framework, two specific reductions that optimally balance the tradeoff between predictive accuracy and any notion of single-criterion definition of fairness were proposed by the authors.

**Figure 6:**
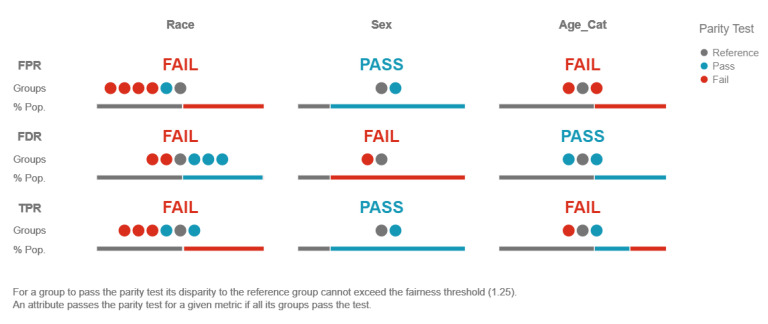
Parity testing, using the aequitas library, on the ProPublica COMPAS Recidivism Risk Assessment dataset, with three metrics: False Positive Rate Disparity, False Discovery Rate Disparity, and True Positive Rate Disparity.

**Figure 7:**
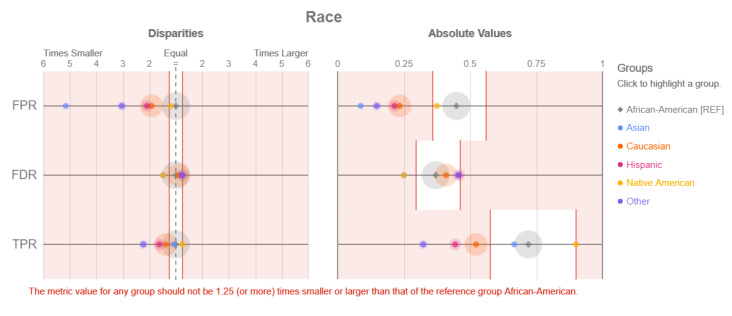
Comparison of the level of race bias (bias disparity) among different groups in the sample population.

In Figure 6 and Figure 7, the use of machine learning interpretability methods to reduce discrimination and promote fairness is presented. More specifically, in Figure 6 parity testing is applied using the aequitas library on the ProPublica COMPAS Recidivism Risk Assessment dataset, whereas in Figure 7, a comparison of the level of race bias (bias disparity) among different groups in the sample population is shown.

In conclusion, fairness is a relatively new domain of machine learning interpretability, yet the progress made in the last few years has been tremendous. Various methods have been created in order to protect disadvantaged demographic segments against social bias and ensure fair allocation of resources. These different methods concern data manipulations prior to model training, algorithmic modifications within the training process as well as post-hoc adjustments. However, most of these methods, regardless of which step of the process they are applied, focus too much on group-level fairness and often ignore individual-level factors both within the groups and at a global scale, potentially mistreating individuals in favour of groups. Furthermore, only a tiny portion of the scientific literature is concerned with fairness in non-tabular data, such as images and text; therefore, a large gap exists in these unexplored areas that are to be filled in the coming years.

### 3.4. Interpretability Methods to Analyse the Sensitivity of Machine Learning Model Predictions

This category includes interpretability methods that attempt to assess and challenge the machine learning models in order to ensure that their predictions are trustworthy and reliable. These methods apply some form of sensitivity analysis, as models are tested with respect to the stability of their learnt functions and how sensitive their output predictions are with respect to subtle yet intentional changes in the corresponding inputs. Sensitivity analysis can be both a global and local interpretation technique, depending on whether the change to the output is analysed with respect to a single example or across all examples in the dataset. Traditional and adversarial example-based sensitivity methods are presented and discussed in this section, while their corresponding summaries are provided in Table 5 and Table 6 respectively.

#### 3.4.1. Traditional Sensitivity Analysis Methods

Traditional sensitivity analysis methods try to represent each input variable with a numeric value, which is called the sensitivity index. Sensitivity indices can be first-order indices, measuring the contribution of a single input variable to the output variance or second, third of higher-order indices, measuring the contribution of the interaction between two, three, or more input variables to the output variance respectively. the total-effect indices, combining the contributions of first-order and higher-order interactions with respect to the output variance.

An output variance sensitivity analysis that is based on the ANOVA decomposition was formalised by Sobol, who proposed the approximation of sensitivity indices of first and higher order while using Monte-Carlo methods [101], while Saltelli [102] and Saltelli et al. [103] improved upon Sobol’s approach while using more efficient sampling techniques for first, higher, as well as total-effect indices.

Cukier et al. [104] proposed the Fourier Amplitude Sensitivity Test (FAST) method to improve the approximation of Sobol’s indices. This is achieved by applying a Fourier transformation to transform a multi-dimensional integral into a one-dimensional integral with different transformations leading to different distributions of sampled points. Saltelli et al. [105] improved upon FAST to compute the total-effect indices, while Tarantola et al. [106] extended random balance designs, applied by Satterthwait in regression problems, to sensitivity analysis for non-linear, non-additive models by combining them with FAST (RBD-FAST). The RBD-FAST method was further improved in terms of computational efficiency by Plischke [107], while Tissot et al. introduced a bias correction method in order to improve estimation accuracy [108].

Another method for global sensitivity analysis is that of Morris [110], which is often referred to as the one-step-at-a-time method (OAT). Under this approach, the input variables are split into three groups: input variables whose contributions are insignificant, inputs that have significant linear effects of their own without any interactions, and inputs that have significant non-linear and/or interaction effects. This is achieved through discretising the input space for each variable and iteratively making a number of local changes (one at a time) at different points for the possible range of input values. The Morris method, while complete, is very costly and, as a result, in some cases, fractional factorial designs, as described in [109], need to be formulated and employed in practice, in order for sensitivity analysis to be performed more efficiently. By devising a more effective sampling strategy as well as other improvements, Campolongo et al. [111] proposed an improved version of Morris’s method.

In some cases, variance is not a good proxy for the variability of the distribution. As a result, some studies have focused on developing sensitivity indices that are not based on variance, which are often referred to as moment independent importance measures, requiring no calculation of the output moments. One example is Borgonovo’s [112] distribution or density based sensitivity indices, which measure the distance or the divergence between the unconditional output distribution and output distribution when conditioned on one or more input variables. Building on from the work of Borgonovo, Plischke et al. [113] introduced a new class of estimators for approximating density-based sensitivity measures, independent of the sampling generation method used.

Being introduced by Sobol and Kucherenko [114], the method of derivative-based global sensitivity measures (DGSM) is based on finding the averages of local derivatives while using Monte Carlo or Quasi Monte Carlo sampling methods. DGSM, which can be seen as the generalization of the Morris method, are much easier to implement and evaluate when compared to the Sobol sensitivity indices.

#### 3.4.2. Adversarial Example-based Sensitivity Analysis

Adversarial examples are datapoints whose features have been perturbed by a subtle yet sufficient amount, enough to cause a machine learning model make incorrect predictions about them. Adversarial examples are like counterfactual examples; however, they do not focus on explaining the model, but on misleading it. Adversarial example-based sensitivity analysis methods are methods that create adversarial examples for different kinds of data such as images or test.

**Figure 8:**
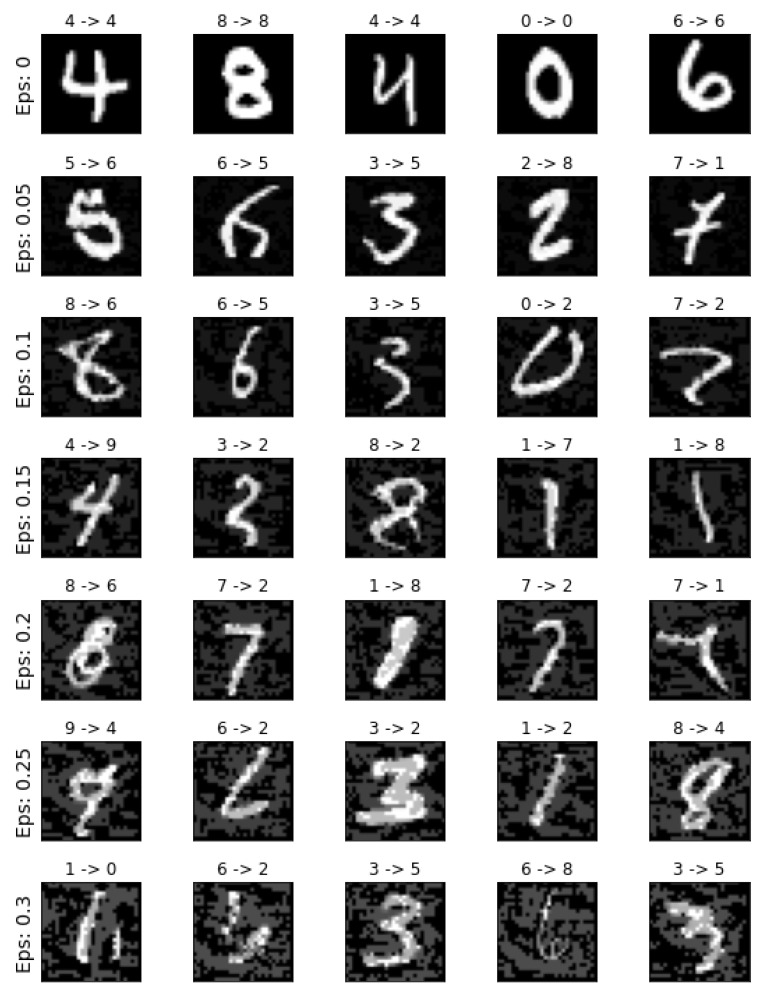
Fast Gradient Sign Attack (FGSM) on the MNIST dataset. The first row contains unperturbed images, while in the subsequent rows are perturbed using some ϵ value, resulting in the model misclassifying them.

It was Szegedy et al. [115] who first discovered that the functions learnt by deep neural networks can be significantly discontinuous, thus their output is very fragile to certain input perturbations. The term “adversarial examples” was coined for such perturbations and it was found that adversarial examples can be shared among neural networks with different architectures, trained on different subsets, disjoint or not, of the same data: the very same input perturbations that caused one network to misclassify can cause a different network to also alter its output dramatically. The problem of finding the minimal necessary perturbations was formulated as a box-constrained L_2_-norm optimisation problem and the L-BFGS optimisation algorithm was employed in order to approximate its solution. Goodfellow et al. [116] argued that high-confidence neural network misclassifications that are caused by small, yet intentionally, worst-case datapoint perturbations, were not due to nonlinearity or overfitting, but instead due to neural networks’ linear nature. In addition to their findings, they also proposed a fast and simple yet powerful gradient-based method of generating adversarial examples while using the L_∞_ norm, called fast gradient sign method (FGSM). Figure 8 illustrates the effectiveness of the FGSM method, where instances of the MNIST dataset are perturbed while using different values of ϵ, resulting in the model misclassifying them.

In order to test the sensitivity of deep learning models, Moosavi-Dezfooli et al. proposed DeepFool [117], a method that generates minimum-perturbation adversarial examples that are optimised for the L_2_ norm. By making the simplifying assumptions, DeepFool employs an iterative process of classifier linearisation, producing adversaries that work well against both binary and multi-class classifiers. Moosavi-Dezfooli et al. [118] also came up with a formulation that is able to produce a single perturbation, such that the classifier mis-classifies most of the instances. The existence of these so called “universal adversarial examples” exposed the inherent weaknesses of deep neural networks on all of the inputs. Papernot et al. [119] conducted a thorough investigation regarding the adversarial behaviour within the deep learning framework and proposed a new class of algorithms able to generate adversarial instances. More specifically, the method exploiting the mathematical relationship between the inputs and outputs of deep neural networks to compute forward derivatives and subsequently construct adversarial saliency maps. Finally, the authors pointed towards the development and utilisation of a distance metric between non-adversarial inputs and the corresponding target labels as a way to defend against adversarial examples. Kurakin et al. [120] highlighted that, although in most studies regarding machine learning sensitivity it is assumed the adversary examples can be input directly into the classifier, this assumption does not always hold true for classifiers engaging with the physical world, such as those receiving input in the form of signals from other devices. To this end, among the other methods used, a new method that improves upon the FGSM [116] algorithm was introduced, whereby the FGSM was repeated many times with small step size, truncating the intermediate results after each step in the process, so that the produced adversarial examples (pixels in this case) are within close range of the original examples. Dong et al. [121] promoted the use of momentum in oder to enhance the process of creating adversarial instances while using iterative algorithms, thus introducing the a broad class of adversarial momentum-based iterative algorithms. Momentum is well known to help iterative optimisation algorithms, such as gradient descent, in order to stabilise gradients and escape from local minima/maxima.

NATTACK: instead of seeking an optimal adversarial example Li et al. [122] considered fitting a probability distribution in a neighbourhood centered around a given example, with the assumption being that any example generated from this distribution is a good adversary candidate. The proposed approach offers two distinct benefits: first, it can be employed to attack any model and, secondly, it does not require of any knowledge of the model’s internal workings.

Carlini and Wagner [123] introduced three novel adversarial attack algorithms, based on the L_0_, L_2_, and L_∞_ norms, respectively, which were very effective against neural networks, even those where defensive distillation technique [124] had been applied. The proposed attacks aim to address the same minimal perturbation problem as Szegedy et al [115], but they formulate it using the margin loss instead of cross-entropy loss, thus minimising the distance between adversarial and benign examples in a more direct way. In [125], Carlini et al. demonstrated how to construct a provable strongest attack, also called the ground truth attack. The problem of finding adversarial examples proven to be of minimal distortion was formulated as a linear-like optimisation problem. The deduced adversarial example, having the most similarity to the original instance, is called the ground truth adversarial example.

Spatially Transformed Attack: Xiao et al. [126] proposed perturbing images by performing a slight spatial transformation such as translating, rotating and/or distorting the image features. Such perturbations are small enough to escape human attention but are able to trick models.

One-pixel Attack: Su et al. [127] showed how neural networks can be fooled by altering the value of just a single input pixel. By constraining the L_0_ norm, they enforced a limit on the number of pixels that were allowed to be perturbed.

Zeroth order optimisation based attack (ZOO): assuming that the one has access to the prediction probability scores (rather than just the predicted labels) of a classifier and the respective inputs, Chen et al. [128] proposed an algorithm to infer the gradient information by observing the changes in the prediction scores, thus eliminating the need for a substitute model when creating adversarial examples.

In their study [129], Narodytska et al. focused on generating adversarial examples for any deep convolutional neural network without prior knowledge of the internal workings of the network in question. To this end, they proposed two pixel-perturbing methods that operate without using any gradient information: the first one is to randomly select and perturb a set of pixels, while the second one improves upon the first one by incorporating a greedy local-search algorithm to efficiently locate a better set of pixels to perturb. Introduced in [130], HopSkipJumpAttack is a group of adversarial-example generating algorithms that rely on binary information regarding the decision boundary and Monte Carlo methods in order to approximate the direction of the gradient. The method is able to produce both targeted and non-targeted examples that are optimised for the L2 and L_∞_ norms.

Liu et al. [131] performed a thorough investigation on the transferability of both non-targeted and targeted adversarial examples while using models and datasets of large scale, concluding that while transferring non-targeted adversarial examples can be very effective in fooling neural networks, targeted adversarial examples do not work as well. To this end, they proposed new ways of producing effective, transferable adversarial examples, both targeted and non-targeted, with a high success rate when tested against a black-box image classification model. Houdini [132] is an approach that was proposed by Cisse et al. that is able to produce adversarial instances for any specified task, according to the respective measure of performance. Houdini’s adversarial attacks were employed with success to a variety of structured prediction tasks, including the typical image classification challenge, but also extending the use of adversarial examples to other problems, such as voice recognition, pose estimation, and semantic segmentation. Finally, it should not be left unnoted that, in terms of measures of performance for the different tasks, Houdini is capable of handling complex measures, even non-decomposable ones as well as combinations of measures. In [133], a novel approach that uses an elastic net-based regularisation framework (the combination of the L_1_ and L_2_ norms) to generate adversarial instances against deep neural networks was proposed. Empirical results on three different image datasets showed that the proposed framework was able to produce adversarial examples that can break through the defensive distillation technique and have high transferablity. Lastly, the inner workings of the method and its way of exploiting the L_1_ norm revealed new useful insights behind the relationship between the L_1_ norm and generation of effective adversarial examples. Papernot et al. [134] proposed a novel method for generating adversarial examples by examining the inputs that were provided to a deep neural network and the corresponding the labels that were assigned by the network. The method consists of training a model using synthetic instances, generated by an adversary, as input and the neural network’s predictions of these instances as the true labels. The trained model is subsequently used to create adversarial examples to attack the neural network. Such examples would be misclassified not only by the trained model, but also by the neural network, as, by definition, they would have similar decision boundaries.

Brendel et al. [135] highlighted the lack of scientific studies regarding decision-based adversarial attacks and pinpointed to the benefits and the versatility of such attacks, namely that they can be used against any black-box model, require only the observing of the model’s final decisions, are easier to implement compared to transfer-based attacks, and, at the same time, are more effective against simple defences when compared to gradient-based or score-based attacks. To support their arguments, they introduced the so-called Boundary Attack, a decision-boundary based adversarial attack, which, in principle, begins with creating adversarial instances of high degree perturbations and, subsequently, decreasing the level of perturbation. More specifically, through a rejection process, the method learns the decision boundary between non-adversarial and adversarial instances and, with this knowledge, is able to generate effective adversaries. Brendel et al. [136] also developed a novel family of gradient-based adversarial attacks that not only performed better than previous gradient-based attacks, but were more effective against gradient-masking, more efficient in terms of querying the defended model, and able to optimise for a variety of adversarial criteria. Unlike other methods that explore areas far away from the decision boundary and, as a result, might get stuck, the point-wise attack only stays in areas close the boundary, where gradient signals are more reliable, in order to minimise the distance between the adversarial and original example. Koh and Liang [137] proposed an indirect method of generating adversarial examples. The proposed method is capable of explicitly calculating what the difference would be in the final loss if one training example was altered without retraining the model. By identifying the training instances with the highest impact on model’s predictions, powerful adversaries can be deducted.

In the works of Zugner et al. [138] and Dai et al. [139], adversarial examples in graph-structured data were studied. The former method is a greedy approach that is concerned with attacking node classification models, through the modification of the node connections (add/remove edges between nodes) or node features (flip the feature of nodes with limited number of operations). Three different settings were considered: manipulation of all nodes in the graph, of a set of nodes, including the node in question, and a set of nodes excluding the node in question. The latter attack method is based on a reinforcement learning formulation of the problem and, more specifically, a Q-Learning game. Under this approach only the addition and removal of edges is allowed when altering the graph structure.

In [140], a graph attack based on meta-learning was proposed. Meta-learning has been historically employed for fast reinforcement learning, hyperparameter tuning, and few-shot image recognition. In this scenario, the graph structure of the network was used as input to a meta-learning algorithm as the hyperparameter to be optimised.

Sharif et al. [141] proposed a method for fooling face recognition neural networks by modifying the original images through the insertion of 3D printed sunglasses in the original face images. The colour of these glasses was optimised towards leading the neural network to mis-classify the faces in question. Hayes and Danezis [142] introduced a generative universal adversarial example framework, whereby image perturbations are produced by a generative model, such that, when incorporated into a normal, non-adversarial instance, they transform it to an adversarial instance. Because the generator is not conditioned on the given images, the generated perturbations can be applied to any image and then transform it into an adversarial one. Schott et al. also developed a high-accuracy, robust against adversarial attacks, image classification model that utilises the analysis by synthesis approach [143]. More specifically, for each instance in the datase, a lower bound of the ELBO loss given each class is calculated and, subsequently, these class-conditional ELBOs are synthesised in order to produce the final prediction. Furthermore, two new attacks were developed: one specifically tailored to work well against the proposed model by exploiting its structure and a decision-based attack that optimises towards the smallest number of perturbed pixels.

In noise-based adversarial attacks, original examples are perturbed with the addition of some form of noise and before being passed as input to a machine learning model. However, in many cases, this addition of noise can cause some input values to fall outside their originally defined domain and therefore clipping is required if they are to passed to the model. The proposed clipping methods prior to [144] were relatively slow and they only provided approximations to the optimal solution, thus diminishing the effectiveness of the produced. adversarial examples. In order to improve both the effectiveness and speed of the previously proposed clipping methods, Rauber and Bethge [144] proposed a fast and differentiable algorithm to rescale perturbation vectors, under which a perturbation with the desired norm after clipping can be analytically calculated while using a closed form solution.

Adversarial example vulnerability also exists in deep reinforcement learning modelling, as demonstrated by Huang et al. [145]. By employing the FGSM method [116], the authors created adversarial states to manipulate the network’s policy. They showed that even slight state perturbations can potentially lead to very significant differences in terms of performance and decisions.

Yang et al. [146] focussed on generating adversarial examples for discrete data such as text. Firstly, a two-step greedy approach that locates which words in a piece of text to perturb and then alters them accordingly was implemented, and, secondly, they proposed a novel method, called Gumbel, where the two steps of the first approach were parameterised and a model was trained to find the optimal ones. Samanta and Mehta [147] as well as Iyyer et al. [148] proposed methods for generating adversarial sentences that are both grammatically correctly and in agreement with the syntax of the original sentences. To this end, the former replaced original words with synonyms and exploited words that, when used in different contexts, have have different meanings, while the latter used paraphrasing techniques. Miyato et al. [149] proposed applying perturbations to the word embeddings in a recurrent neural network instead of the original input. The produced word embeddings were shown to be of greater quality, while the resulting model was shown to be less prone to over-fitting. Ebrahimi et al. [150] considered replacing a single character in a sentence in order to fool character-based text classifiers. Using gradient information, the method identifies the most influential letter to be replaced. A closely related work [151] by Liang et al. creates adversaries by adding, removing, and altering words or phrases instead of single characters. Such words or phrases are identified as more or less influential based on the influence of their individual characters, similarly to [150].

In their study, Jia and Liang [152] investigated generating examples for reading comprehension tasks: given a paragraph and a related question, the model has to generate an answer. Focusing on models while using the Stanford Question Answering Dataset (SQuAD), they devised two attacks, ADDSENT and ADDANY, which both try to create adversarial examples by adding words from the original question. In addition, two variants of the original attacks were developed: ADDONESENT, where a random human-approved sentence is added to the original paragraph, and ADDCOMMON, which is identical to ADDANY, except that common words are added instead. Alzantot et al. [153] proposed a method to generate adversarial examples for text while using a population-based genetic algorithm. The algorithm, operating by looping through every word in each sentence applying perturbations based on swapping counter-fitted word embeddings, yielded very high success rates when its adversarial examples were used to attack sentiment analysis models as well as textual entailment models. A similar idea was later also proposed by Kuleshov et al. [154], which uses word replacement by greedy heuristics, while later Wang et al. [155] improved upon the genetic algorithm, achieving not only higher success rates, but also lower word substitution rates and more transferable adversarial examples when compared to [153].

DeepWordBug: the basic idea behind DeepWordBug [156] is to come up with a scoring strategy that is able to determine those text pieces, which, if manipulated, are most likely to force a model into mis-classifications. Such maniupulations include token insertions, deletions, substitutions as well as k-nearest neighbour token swaps based on cosine similarity. Textbugger [157] works in similar fashion, providing improvements over DeepWordBug through the introduction of novel scoring functions.

Seq2Sick: Cheng et al. [158] considered adversarial attacks against seq2seq models, which were widely adopted in text summarisation and neural machine translation tasks. The two main challenges in producing successful seq2seq attacks include the discrete input domain and the almost infinite output domain. The former problem was addressed through the development of a projected gradient method that combines the regularization method with group lasso, while the latter was handled by using newly-proposed loss functions.

Feng et al. [159] introduced a process, called “input reduction”, which can expose issues regarding overconfidence and oversensitivity in natural language processing models. Under input reduction, non-important words are removed from the input text in interative fashion, while the model’s prediction for that input remains unchanged. The authors demonstrated that input texts can have their words removed to a degree where they make no sense to humans, without any impact on the model’s output. Ren et al. [160] proposed a greedy algorithm for textual adversarial example generation , alled probability weighted word saliency (PWWS), which follows the synonyms substitution strategy, but replaces words that are based on the word saliency and classification probability TextFooler [161] generates adversarial examples for text by utilising word embedding distance and part-of-speech matching to first identify the most important words in terms of the model’s output and subsequently greedily replaces them with synonyms that fit both semantically and grammatically until a mis-classification occurs. The BERT language model was utilised in two studies in order to create textual adversarial examples: Garg and Ramakrishnan [162] and Li et al. [163], both of which proposed generating adversarial examples through text perturbations that are based on the BERT masked language model, as part of the original text is masked and alternative text pieces are generated to replace these masks. In their work [164], Tan et al. proposed Morpheus, which is a method for generating textual adversarial examples by greedily perturbing the inflections of the original words in the text to find the inflected forms with the greatest loss increase, only taking into considerations the inflections that belong to the same part of speech as the original word. Unlike most work on textual adversarial examples, Morpheus produces its adversaries by exploiting the morphology of the text. Zang et al. [165] suggested applying word substitutions using the minimum semantic units, called sememes. The assumption was that the sememes of a word are indicative of the word’s meaning and, therefore, words with the same sememes should be good substitutes for each another. To search for such words efficiently, an algorithm based on particle swarm optimization (PSO) was proposed.

Studies on sensitivity analysis over the recent years have focussed on exposing the weaknesses of deep learning models and their vulnerability against adversarial attacks. The literature is very complete when it comes to fooling models in computer vision and natural language processing tasks. However, minimal work has been done in terms of tabular data—in theory, some of the adversarial example generation techniques from computer vision could be applied to tabular data, but their effectiveness has not yet been clearly demonstrated.

## 4. Discussion and Conclusions

The main contribution of this study is a taxonomy of the existing machine learning interpretability methods that allows for a multi-perspective comparison among them. Under this taxonomy, four major categories for interpretability methods were identified: methods for explaining complex black-box models, methods for creating white-box models, methods that promote fairness and restrict the existence of discrimination, and, lastly, methods for analysing the sensitivity of model predictions.

As a result of the high attention that is paid by the research community to deep learning, the literature around interpretability methods has been largely dominated by neural networks and their applications to computer vision and natural language processing. Most interpretability methods for explaining deep learning models refer to image classification and produce saliency maps, highlighting the impact of the different image regions. In many cases, this is achieved through exploiting the gradient information flowing through the layers of the network, Grad-CAM [35], a direct extension of [34], being a prime and most influential example in terms of citations per year. Another way of creating saliency maps, and the most influential overall while using the same metric, is through the adoption of deconvolutional neural networks [32]. In terms of explaining any black-box model, the LIME [45] and SHAP [48] methods are, by far, the most comprehensive and dominant across the literature methods for visualising feature interactions and feature importance, while Friedman’s PDPs [59], although much older and not as sophisticated, still remains a popular choice. The LIME and SHAP methods are not only model-agnostic, but they have been demonstrated to be applicable to any type of data.

White-box highly performing models are very hard to create, especially in computer vision and natural language processing, where the gap in performance against deep learning models is unbridgeable. Furthermore, because models are more than ever expected to be competitive on more than one tasks and knowledge transfer from one domain to another is becoming a recurring theme, white-box models, being able to perform well only in a single given task, are losing traction within the literature and are quickly falling further behind in terms of interest. The most notable work in this category is that of Caruana et al. [65], who proposed a version of generalized additive models with pairwise interactions (GA^2^ Ms), originally proposed in [66], reporting state-of-the-art accuracy in two healthcare applications.

A great deal of effort and progress has been made towards tackling discrimination and supporting fairness in machine learning that sensitive domains, like banking, healthcare, or law, could benefit from. However, these methods are neither commonly found, nor well-promoted within the dominant machine learning frameworks. In this category, the work of Hardt et al. [92], introducing a generalised framework for quantifying and reducing discrimination in any supervised learning setting, has been a milestone and the point of reference for many other studies. That being said, only few studies deal with fairness in non-tabular data, such as images and text, which leaves plenty of room for improvements and innovation in these unexplored areas in the coming years.

Sensitivity analysis, which is the last category of intepretability methods under this taxonomy, has seen tremendous growth over the past several years following the breakthrough work of Szegedy et al. [115] on adversarial examples and the weaknesses of deep learning models against adversarial attacks. Numerous methods for producing adversarial examples have been developed, with some of them focusing on a more general setting, while others being tailored to specific data types, such as image, text, or even graph data, and to specific learning tasks, such as reading comprehension or text generation.

Despite its rapid growth, explainable artificial intelligence is still not a mature and well established field, often suffering from a lack of formality and not well agreed upon definitions. Consequently, although a great number of machine learning interpretability techniques and studies have been developed in academia, they rarely form a substantial part of machine learning workflows and pipelines.

The volume of studies on machine learning interpretability methods over the past years demonstrated the room for improvement that exists by showcasing the benefits and enhancements that these methods can bring to existing machine learning workflows, but also exposed their flaws, weaknesses, and how much they lack performance-aside. In any case, it is our belief that explainable artificial intelligence still has unexplored aspects and a lot of potential to unlock in the coming years.

## Figures and Tables

**Table 1:** Interpretability Methods to Explain Deep Learning Models.

Ref	Tool	Category	Local vs. Global	Model Specific vs. Model Agnostic	Data Type	Citations/Year	Year
[32]	DeepExplain iNNvestigate tf-explain	PH	L	Specific	img	1548.3	2014
[35]	Grad-CAM tf-explain	PH	L	Specific	img	797.8	2017
[34]	CAM	PH	L	Specific	img	607.8	2016
[31]	iNNvestigate	PH	L	Specific	img	365.3	2014
[23]	DeepExplain iNNvestigate tf-explain	PH	L	Specific	img	278.3	2013
[27]	DeepExplain iNNvestigate Integrated Gradients tf-explain alibi Skater	PH	L	Specific	img txt tab	247	2017
[40]	Deep Visualization Toolbox	PH	L	Specific	img	221.7	2015
[37]	DeepExplain iNNvestigate The LRP Toolbox Skater	PH	L	Specific	img txt	217.8	2015
[29]	DeepExplain DeepLift iNNvestigate tf-explain Skater	PH	L	Specific	img	211.5	2017
[41]	iNNvestigate	PH	L	Specific	img	131.5	2017
[38]	iNNvestigate tf-explain	PH	L	Specific	img	113.3	2017
[42]	tcav	PH	L	Specific	img	95	2018
[43]	rationale	PH	L	Specific	txt	81.4	2016
[36]	Grad-CAM++	PH	L	Specific	img	81	2018
[39]	RISE	PH	L	Specific	img	43.3	2018
[44]	iNNvestigate	PH	L	Specific	img	41.8	2017

**Table 2:** Interpretability Methods to Explain any Black-Box Model.

Ref	Tool	Category	Local vs. Global	Model Specific vs. Model Agnostic	Data Type	Citations/Year	Year
[45]	lime Eli5 InterpretML AIX360 Skater	PH	L	Agnostic	img txt tab	845.6	2016
[59]	PDPbox InterpretML Skater	PH	G	Agnostic	tab	589.2	2001
[48]	shap alibi AIX360 InterpretML	PH	L & G	Agnostic	img txt tab	504.5	2017
[50]	alibi Anchor	PH	L	Agnostic	img txt tab	158.3	2018
[53]	alibi	PH	L	Agnostic	tab img	124.5	2017
[60]	PyCEbox	PH	L & G	Agnostic	tab	53.3	2015
[58]	L2X	PH	L	Agnostic	img txt tab	50.3	2018
[57]	Eli5	PH	G	Agnostic	tab	41.5	2010
[51]	alibi AIX360	PH	L	Agnostic	tab img	34.3	2018
[61]	Alibi	PH	G	Agnostic	tab	23.2	2016
[54]	alibi	PH	L	Agnostic	tab img	17	2019
[62]	pyBreakDown	PH	L	Agnostic	tab	8.3	2018
[62]	pyBreakDown	PH	G	Agnostic	tab	8.3	2018
[47]	DLIME	PH	L	Agnostic	img txt tab	7.5	2019
[56]	AIX360	PH	L	Agnostic	tab	7	2019
[52]	AIX360	PH	L	Agnostic	tab img	3	2019

**Table 3:** Interpretability Methods to Create White-Box Models.

Ref	Tool	Category	Local vs. Global	Model Specific vs. Model Agnostic	Data Type	Citations/Year	Year
[65]	InterpretML	W	G	Specific	tab	129.5	2015
[64]	Slim	W	G	Specific	tab	35.2	2016
[68]	AIX360	W	G	Specific	tab	12.3	2018
[71]	AIX360	W	L	Specific	tab	12	2019
[69]	AIX360	W	G	Specific	tab	5	2019

**Table 4:** Interpretability Methods to Restrict Discrimination and Enhance Fairness in Machine Learning Models.

Ref	Tool	Category	Local vs. Global	Model Specific vs. Model Agnostic	Data Type	Citations/Year	Year
[92]	equalized_odds_and_calibration fairlearn AIF360	F	G	Agnostic	tab	242.2	2016
[85]	debiaswe	F	L	Specific	txt	216.8	2016
[88]	fairness	F	L	Agnostic	tab	133.4	2012
[72]	Aequitas AIF360 themis-ml	F	G	Agnostic	tab	124.5	2015
[93]	fair-classification	F	G	Agnostic	tab	117.8	2017
[84]	fairness-in-ml	F	L	Agnostic	tab	115.5	2017
[94]	fair-classification	F	G	Agnostic	tab	110.8	2017
[86]	AIF360	F	L & G	Agnostic	tab	94.6	2013
[95]	fairlearn	F	G	Agnostic	tab	94	2018
[77]	AIF360	F	L & G	Agnostic	tab	92.3	2018
[96]	AIF360 GerryFair	F	G	Agnostic	tab	76	2018
[97]	equalized_odds_and_calibration AIF360	F	G	Agnostic	tab	60	2017
[76]	AIF360	F	G	Agnostic	tab	53.5	2017
[79]	ML-fairness-gym	F	G	Agnostic	tab	51.7	2018
[81]	ML-fairness-gym	F	G	Agnostic	tab	45.7	2018
[87]	fairness-comparison	F	G	Specific	tab	45	2010
[73]	AIF360	F	G	Agnostic	tab	37.2	2012
[98]	AIF360	F	G	Agnostic	tab	37	2019
[99]	AIF360	F	G	Agnostic	tab	35.3	2012
[100]	fair-classification	F	G	Agnostic	tab	26.8	2017
[83]	ML-fairness-gym	F	L	Specific	tab	24	2019
[82]	ML-fairness-gym	F	L	Specific	tab	23	2019
[89]	procedurally_fair_learning	F	G	Agnostic	tab	22	2018
[74]	themis-ml	F	L	Agnostic	tab	19.6	2009
[75]	AIF360	F	L	Agnostic	tab	17	2009
[78]	AIF360 themis-ml	F	G	Agnostic	tab	12.1	2012
[80]	ML-fairness-gym	F	G	Specific	tab	10.5	2019
[91]	FairMachineLearning	F	L	Specific	tab	3.8	2016

**Table 5:** Traditional Sensitivity Analysis Methods.

Ref	Tool	Category	Local vs. Global	Model Specific vs. Model Agnostic	Data Type	Citations/Year	Year
[109]	SALib	S	G	Agnostic	tab	400.8	2008
[103]	SALib	S	G	Agnostic	tab	160.2	2010
[101]	SALib	S	G	Agnostic	tab	152.4	2001
[110]	SALib	S	G	Agnostic	tab	117.6	1991
[111]	SALib	S	G	Agnostic	tab	101.5	2007
[105]	SALib	S	G	Agnostic	tab	87.5	1999
[102]	SALib	S	G	Agnostic	tab	76.2	2002
[112]	SALib	S	G	Agnostic	tab	50.2	2007
[113]	SALib	S	G	Agnostic	tab	29.9	2013
[114]	SALib	S	G	Agnostic	tab	29.1	2009
[104]	SALib	S	G	Agnostic	tab	21.6	1973
[106]	SALib	S	G	Agnostic	tab	16.5	2006
[107]	SALib	S	G	Agnostic	tab	9.2	2010
[108]	SALib	S	G	Agnostic	tab	6.3	2012

**Table 6:** Adversarial Example-based Sensitivity Analysis.

Ref	Tool	Category	Local vs. Global	Model Specific vs. Model Agnostic	Data Type	Citations/Year	Year
[116]	cleverhans foolbox	S	L & G	Agnostic	img	876.4	2014
[115]	cleverhans foolbox	S	L & G	Agnostic	img	727.4	2013
[123]	cleverhans nn_robust_attacks	S	L & G	Agnostic	img	716	2017
[120]	cleverhans foolbox	S	L & G	Agnostic	img	429	2016
[127]	one-pixel-attack-keras	S	L & G	Agnostic	img	409	2019
[117]	cleverhans foolbox	S	L & G	Agnostic	img	392	2016
[119]	cleverhans foolbox	S	L & G	Agnostic	img	381.2	2016
[134]	cleverhans	S	L & G	Agnostic	img	378.8	2017
[137]	influence-release	S	L & G	Agnostic	img	224	2017
[121]	cleverhans	S	L & G	Agnostic	img	181.7	2018
[152]	adversarial-squad	S	L & G	Specific	txt	162	2017
[131]	transferability-advdnn-pub	S	L & G	Agnostic	img	148.6	2016
[141]	accessorize-to-a-crime	S	L & G	Agnostic	img	141.6	2016
[128]	ZOO-Attack	S	L & G	Agnostic	img	129.8	2017
[135]	foolbox boundary-attack	S	L & G	Agnostic	img	99.5	2017
[158]	TextAttack	S	L & G	Specific	txt	83	2020
[149]	adversarial_text adversarial_training adversarial_training_methods	S	L & G	Specific	txt	70.8	2016
[138]	nettack	S	L & G	Specific	graph data	70.3	2018
[153]	nlp_adversarial_examples	S	L & G	Agnostic	txt	70.3	2018
[148]	scpn	S	L & G	Agnostic	txt	66.7	2018
[126]	stAdv	S	L & G	Agnostic	img	65.3	2018
[133]	cleverhans	S	L & G	Agnostic	img	64.3	2017
[150]	WordAdver	S	L & G	Agnostic	txt	63.5	2017
[139]	graph_adversarial_attack	S	L & G	Specific	graph data	55.3	2018
[143]	foolbox AnalysisBySynthesis	S	L & G	Agnostic	img	44.3	2018
[140]	gnn-meta-attack	S	L & G	Specific	graph data	42	2019
[156]	TextAttack	S	L & G	Agnostic	txt	41	2018
[118]	universal	S	L & G	Agnostic	img	34	2017
[159]	TextAttack	S	L & G	Agnostic	txt	31.7	2018
[130]	HSJA	S	L & G	Agnostic	img	31.5	2019
[160]	TextAttack	S	L & G	Agnostic	txt	29.5	2019
[122]	Nattack	S	L & G	Agnostic	img	29	2019
[129]	foolbox	S	L & G	Agnostic	img	26.8	2016
[157]	TextAttack	S	L & G	Agnostic	txt	26.7	2018
[161]	TextAttack TextFooler	S	L & G	Specific	txt	21	2019
[125]	nn_robust_attacks	S	L & G	Agnostic	img	11	2017
[165]	TextAttack	S	L & G	Agnostic	txt	10	2020
[142]	UAN	S	L & G	Agnostic	img	9.7	2018
[154]	TextAttack	S	L & G	Agnostic	txt	9.3	2018
[136]	foolbox	S	L & G	Agnostic	img	6.5	2019
[155]	TextAttack	S	L & G	Agnostic	txt	6.5	2019
[163]	TextAttack	S	L & G	Specific	txt	5	2020
[164]	TextAttack	S	L & G	Agnostic	txt	5	2020
[162]	TextAttack	S	L & G	Specific	txt	4	2020
[144]	foolbox	S	L & G	Agnostic	img	1.5	2019

## Abbreviations

The following abbreviations are used in this manuscript:

W	Whit-Box/Interpretable Models
PH	Post-Hoc
F	Fairness
S	Sensitivity
L	Local
G	Global
Agnostic	Model Agnostic
Specific	Model Specific
tab	Tabular Data
img	Image Data
txt	Text Data
graph	Graph Data

## Appendix A. Repository Links

**Table A1:** Repository Links.

Tool	Repository Link
accessorize-to-a-crime	https://github.com/mahmoods01/accessorize-to-a-crime
adversarial-squad	https://github.com/robinjia/adversarial-squad
adversarial_text	https://github.com/aonotas/adversarial_text
adversarial_training	https://github.com/WangJiuniu/adversarial_training
adversarial_training_methods	https://github.com/enry12/adversarial_training_methods
aequitas	https://github.com/dssg/aequitas
AIF360	https://github.com/Trusted-AI/AIF360
AIX360	https://github.com/Trusted-AI/AIX360
alibi	https://github.com/SeldonIO/alibi
AnalysisBySynthesis	https://github.com/bethgelab/AnalysisBySynthesis
Anchor	https://github.com/marcotcr/anchor
boundary-attack	https://github.com/greentfrapp/boundary-attack
CAM	https://github.com/zhoubolei/CAM
cleverhans	https://github.com/tensorflow/cleverhans
debiaswe	https://github.com/tolga-b/debiaswe
Deep Visualization Toolbox	https://github.com/yosinski/deep-visualization-toolbox
DeepExplain	https://github.com/marcoancona/DeepExplain
DeepLift	https://github.com/kundajelab/deeplift
DLIME	https://github.com/rehmanzafar/dlime_experiments
Eli5	https://github.com/TeamHG-Memex/eli5
equalized_odds_and_calibration	https://github.com/gpleiss/equalized_odds_and_calibration
fair-classification	https://github.com/mbilalzafar/fair-classification
fairlearn	https://github.com/fairlearn/fairlearn
FairMachineLearning	https://github.com/jtcho/FairMachineLearning
fairml	https://github.com/adebayoj/fairml
fairness	https://github.com/dodger487/fairness
fairness-comparison	https://github.com/algofairness/fairness-comparison
fairness-in-ml	https://github.com/equialgo/fairness-in-ml
foolbox	https://github.com/bethgelab/foolbox
GerryFair	https://github.com/algowatchpenn/GerryFair
gnn-meta-attack	https://github.com/danielzuegner/gnn-meta-attack
Grad-CAM	https://github.com/ramprs/grad-cam
Grad-CAM ++	https://github.com/adityac94/Grad_CAM_plus_plus
graph_adversarial_attack	https://github.com/Hanjun-Dai/graph_adversarial_attack

**Table A2:** Repository Link (2).

Tool	Repository Link
HSJA	https://github.com/Jianbo-Lab/HSJA
influence-release	https://github.com/kohpangwei/influence-release
iNNvestigate	https://github.com/albermax/innvestigate
Integrated Gradients	https://github.com/ankurtaly/Integrated-Gradients
InterpretML	https://github.com/interpretml/interpret
L2X	https://github.com/Jianbo-Lab/L2X
lime	https://github.com/marcotcr/lime
ML-fairness-gym	https://github.com/google/ml-fairness-gym
Nattack	https://github.com/gaussian-attack/Nattack
nettack	https://github.com/danielzuegner/nettack
nlp_adversarial_examples	https://github.com/nesl/nlp_adversarial_examples
nn_robust_attacks	https://github.com/carlini/nn_robust_attacks
one-pixel-attack-keras	https://github.com/Hyperparticle/one-pixel-attack-keras
PDPbox	https://github.com/SauceCat/PDPbox
procedurally_fair_learning	https://github.com/nina-gh/procedurally_fair_learning
pyBreakDown	https://github.com/MI2DataLab/pyBreakDown
PyCEbox	https://github.com/AustinRochford/PyCEbox
rationale	https://github.com/taolei87/rcnn/tree/master/code/rationale
RISE	https://github.com/eclique/RISE
scpn	https://github.com/miyyer/scpn
SALib	https://github.com/SALib/SALib
shap	https://github.com/slundberg/shap
Skater	https://github.com/oracle/Skater
Slim	https://github.com/ustunb/slim-python
stAdv	https://github.com/rakutentech/stAdv
tcav	https://github.com/tensorflow/tcav
TextAttack	https://github.com/QData/TextAttack
TextFooler	https://github.com/jind11/TextFooler
tf-explain	https://github.com/sicara/tf-explain
The LRP Toolbox	https://github.com/sebastian-lapuschkin/lrp_toolbox
themis-ml	https://github.com/cosmicBboy/themis-ml
transferability-advdnn-pub	https://github.com/sunblaze-ucb/transferability-advdnn-pub
UAN	https://github.com/jhayes14/UAN
universal	https://github.com/LTS4/universal
WordAdver	https://github.com/AnyiRao/WordAdver
ZOO-Attack	https://github.com/huanzhang12/ZOO-Attack
